# Supplementary material for: Zimin patterns in genomes
Source: PLoS Comput Biol. 2026 Feb 9;22(2):e1013909. doi: 10.1371/journal.pcbi.1013909 (PMC12912701; doi:10.1371/journal.pcbi.1013909)
Supplement: S1 File — Fig A: Relationship of GC-Content and Zimin avoidmer density across the simulated genomes of human telomere-to-telomere reference genome. For each simulated genome, the genome-wide avoidmer density was evaluated, and a second degree linear regression was used to model the relationship between the GC-content and avoidmer density. Fig B: Fold enrichment of avoidmers in simulated genomes of the human telomere-to-telomere reference genome. A) Fold enrichment of avoidmer density of simulated shuffled genomes with respect to the reference genome, displayed in increasing order of preserved nucleotide composition starting from mononucleotides up to nine-nucleotides. The significance of the enrichment was evaluated using one-tailed binomial test, with background base rate being chosen as the the probability of a single base pair belonging to an avoidmer sequence of at least 50 bp long, the number of positive events equal to the number of avoidmer base pairs in the shuffled genome, and the genome size as the total number of iterations. B) Fold enrichment of avoidmer density of simulated shuffled genomes with respect to the human reference genome, as a function of the preserved nucleotide composition, for various minimum avoidmer thresholds. C) Maximum avoidmer length increase across shuffled genomes. Fig C: Avoidmer density across the Y chromosome. The highlighted regions correspond to the classical human satellite region hsat1B where the vast majority of avoidmers are located. The densities of Zimin avoidmer sequences of at least 70 bp long, appear denser in the hsat1B compartments rather than the hsat3 satellite regions. Fig D: ABA sub-sequence density in avoidmers and its relationship with canonical k-mer diversity. A. ABA sub-sequence density in avoidmer sequences. B. ABA density in avoidmers as a function of average canonical k-mer diversity for various k-mer lengths. Fig E: Kernel density estimation of k-mer diversity scores of 5kB, 10kB, 25kB, 100kB regions that contai [file pcbi.1013909.s001.docx]

**Supplementary Material**


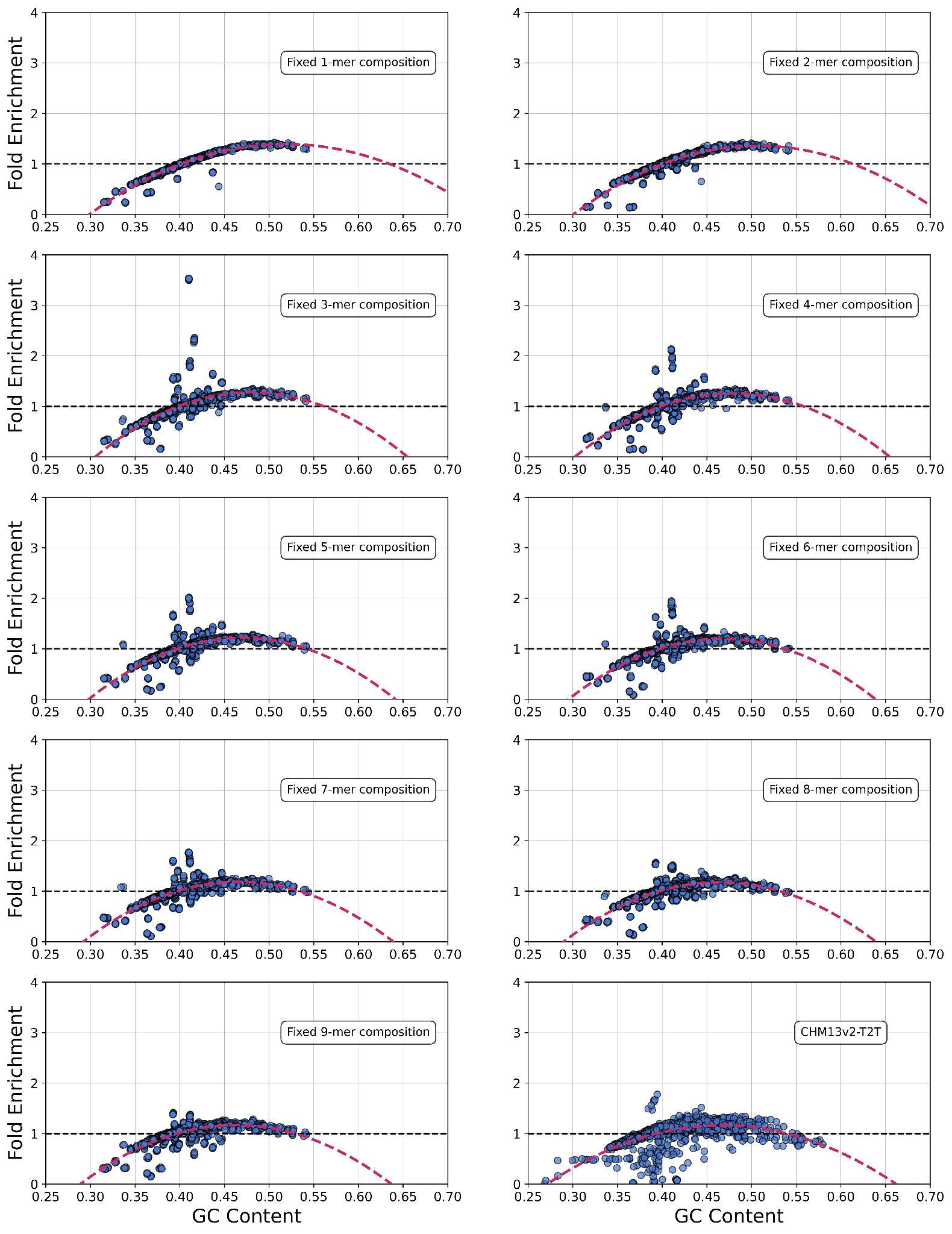


**Fig A. Relationship of GC-Content and Zimin avoidmer density across the simulated genomes of human telomere-to-telomere reference genome.** For each simulated genome, the genome-wide avoidmer density was evaluated, and a second degree linear regression was used to model the relationship between the GC-content and avoidmer density.


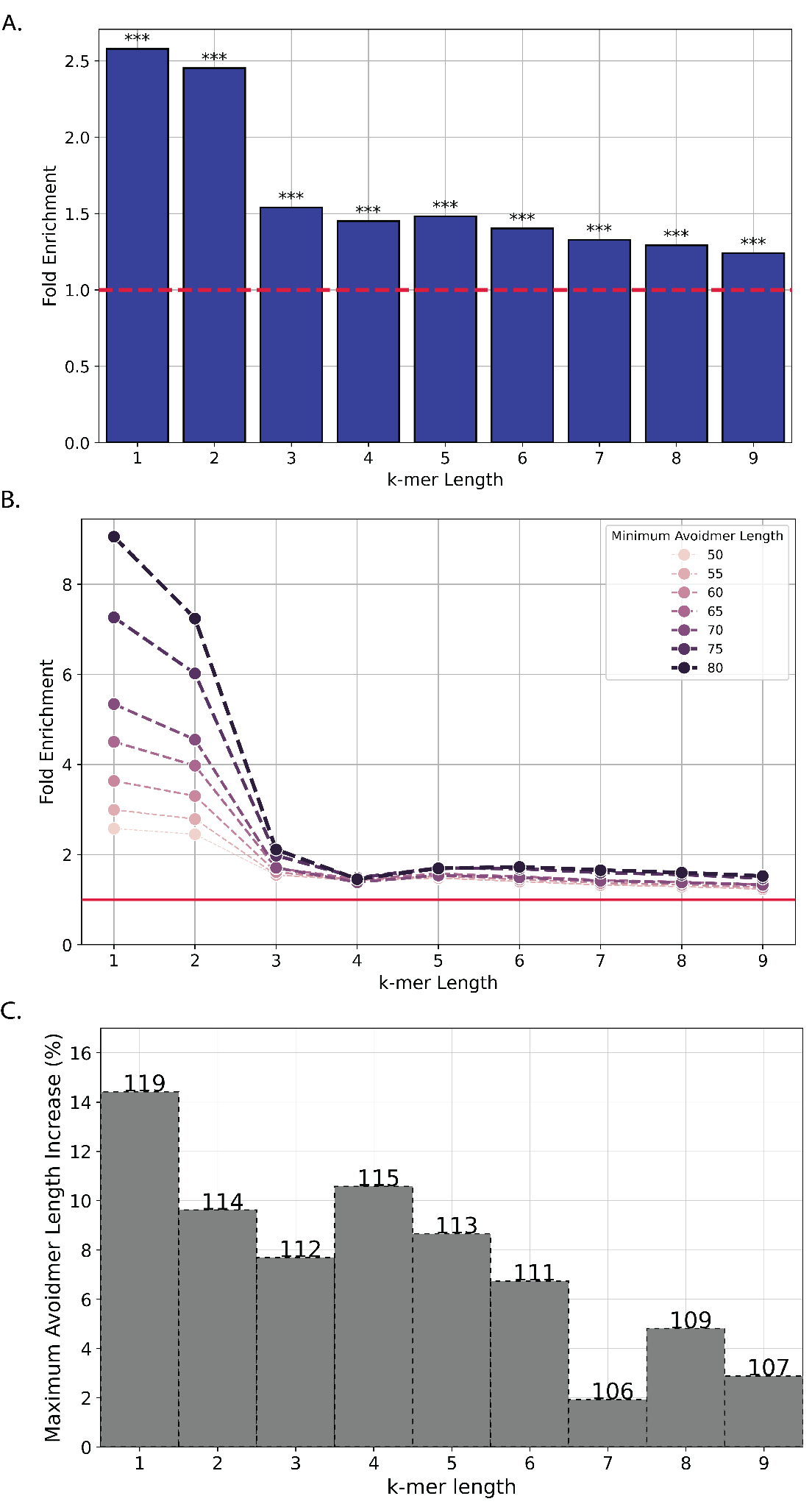


**Fig B. Fold enrichment of avoidmers in simulated genomes of the human telomere-to-telomere reference genome**. **A)** Fold enrichment of avoidmer density of simulated shuffled genomes with respect to the reference genome, displayed in increasing order of preserved nucleotide composition starting from mononucleotides up to nine-nucleotides. The significance of the enrichment was evaluated using one-tailed binomial test, with background base rate being chosen as the the probability of a single base pair belonging to an avoidmer sequence of at least 50bp long, the number of positive events equal to the number of avoidmer base pairs in the shuffled genome, and the genome size as the total number of iterations. **B)** Fold enrichment of avoidmer density of simulated shuffled genomes with respect to the human reference genome, as a function of the preserved nucleotide composition, for various minimum avoidmer thresholds. **C)** Maximum avoidmer length increase across shuffled genomes.


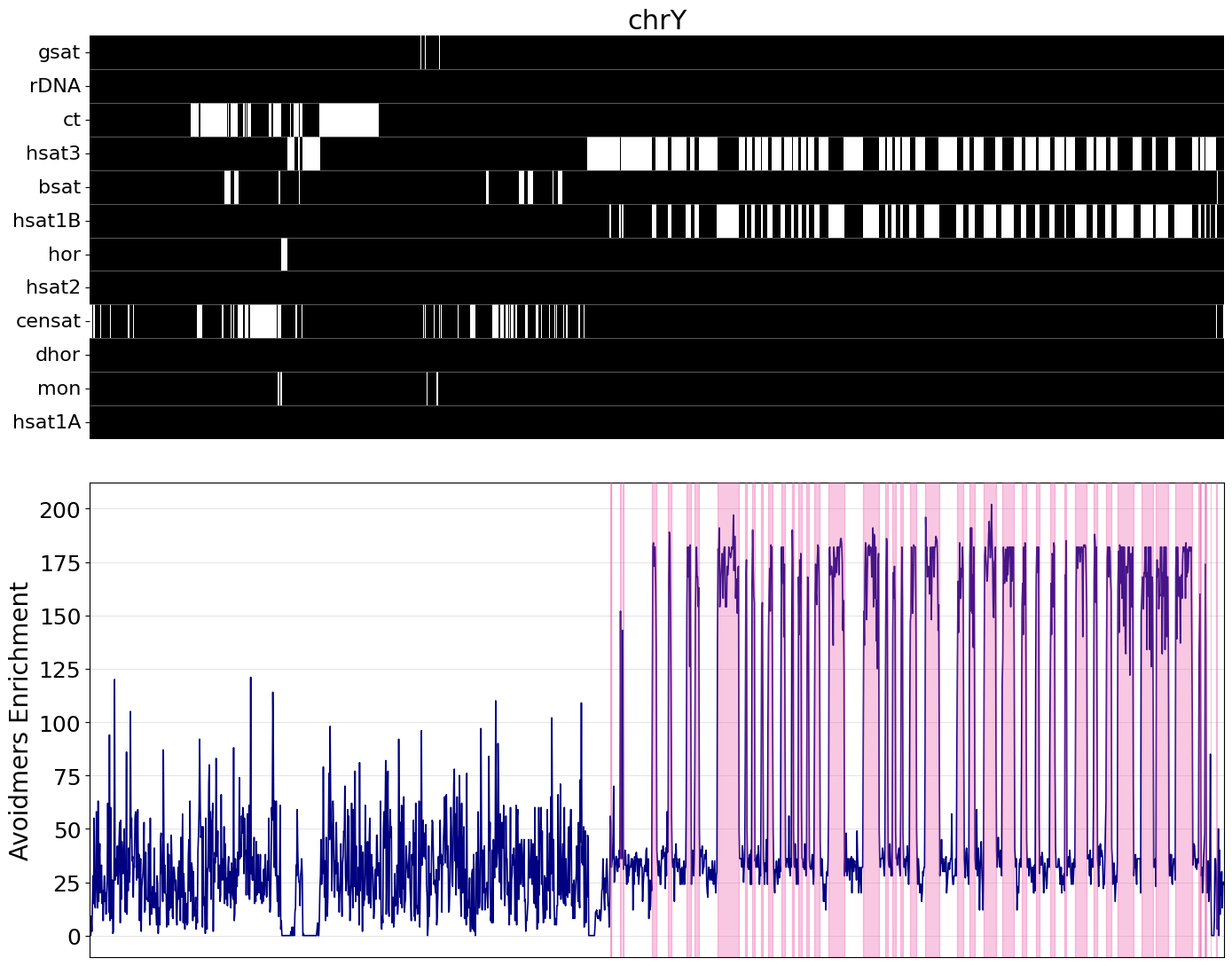


**Fig C. Avoidmer density across the Y chromosome.** The highlighted regions correspond to the classical human satellite region hsat1B where the vast majority of avoidmers are located. The densities of Zimin avoidmer sequences of at least 70 bp long, appear denser in the hsat1B compartments rather than the hsat3 satellite regions.


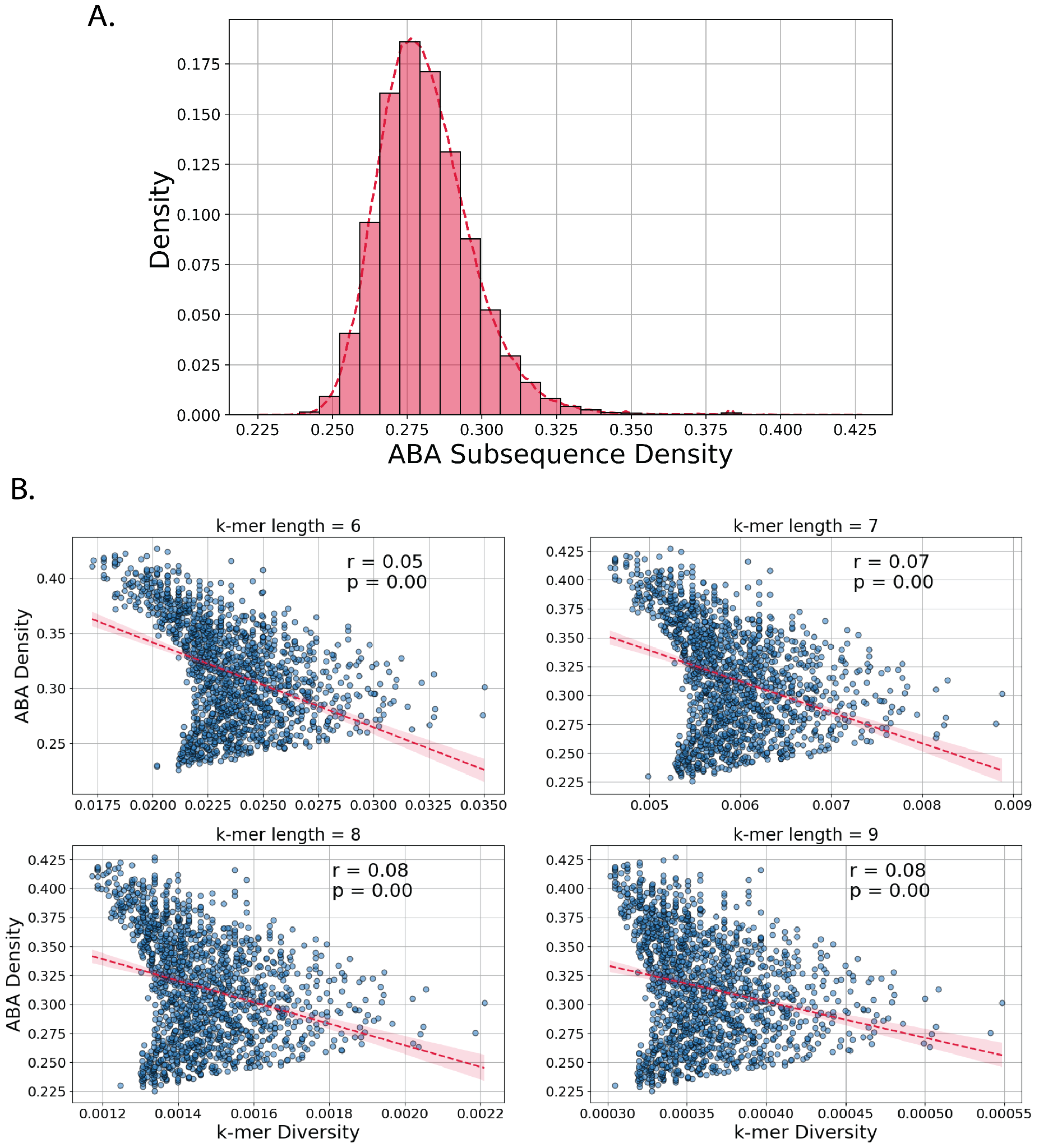


**Fig D. ABA sub-sequence density in avoidmers and its relationship with canonical k-mer diversity. A.** ABA sub-sequence density in avoidmer sequences. **B.** ABA density in avoidmers as a function of average canonical k-mer diversity for various k-mer lengths.


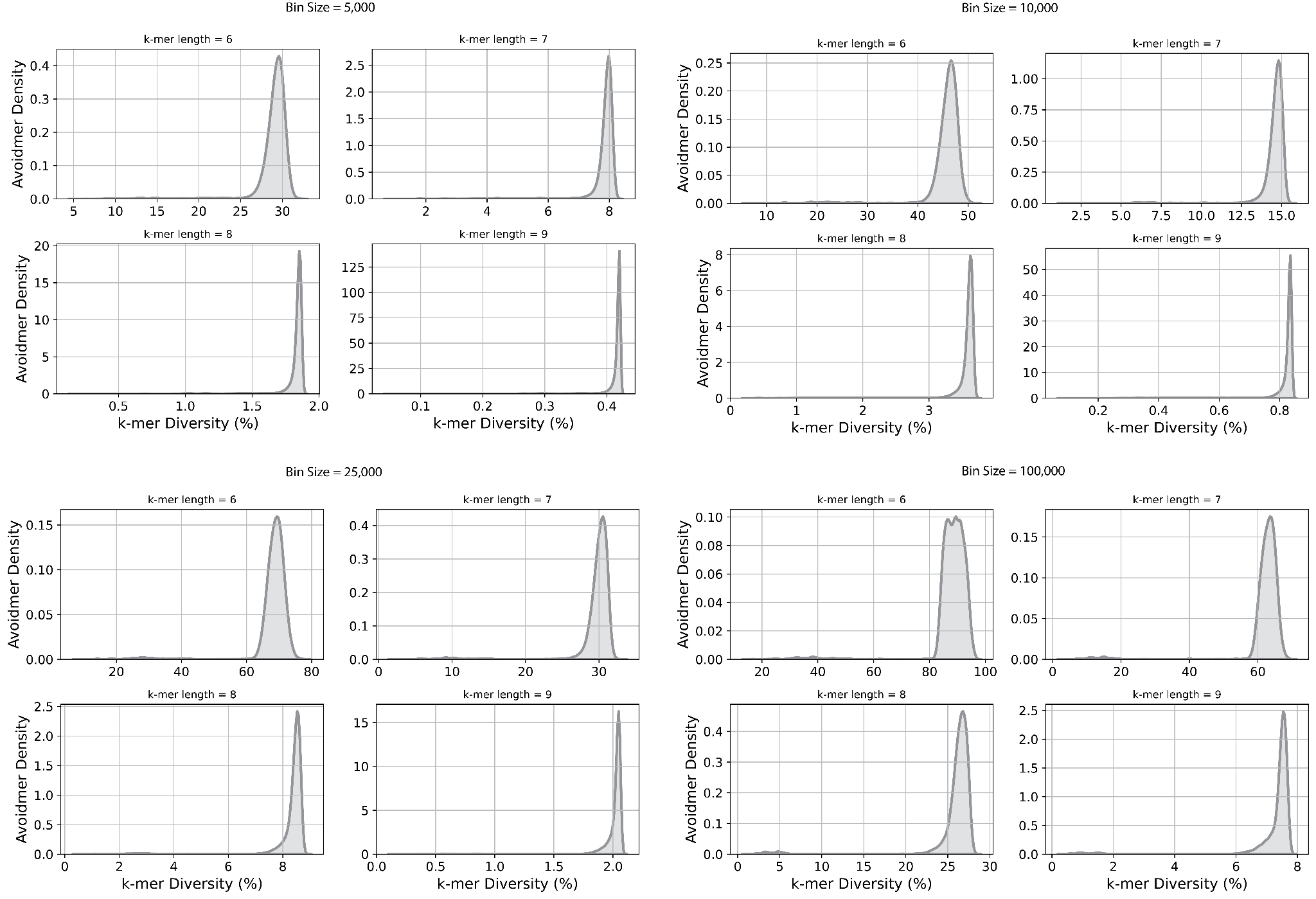


**Fig E. Kernel density estimation of k-mer diversity scores of 5kB, 10kB, 25kB, 100kB regions that contain a Zimin avoidmer of at least 50bp long, for k-mer lengths between six and nine base-pairs.**


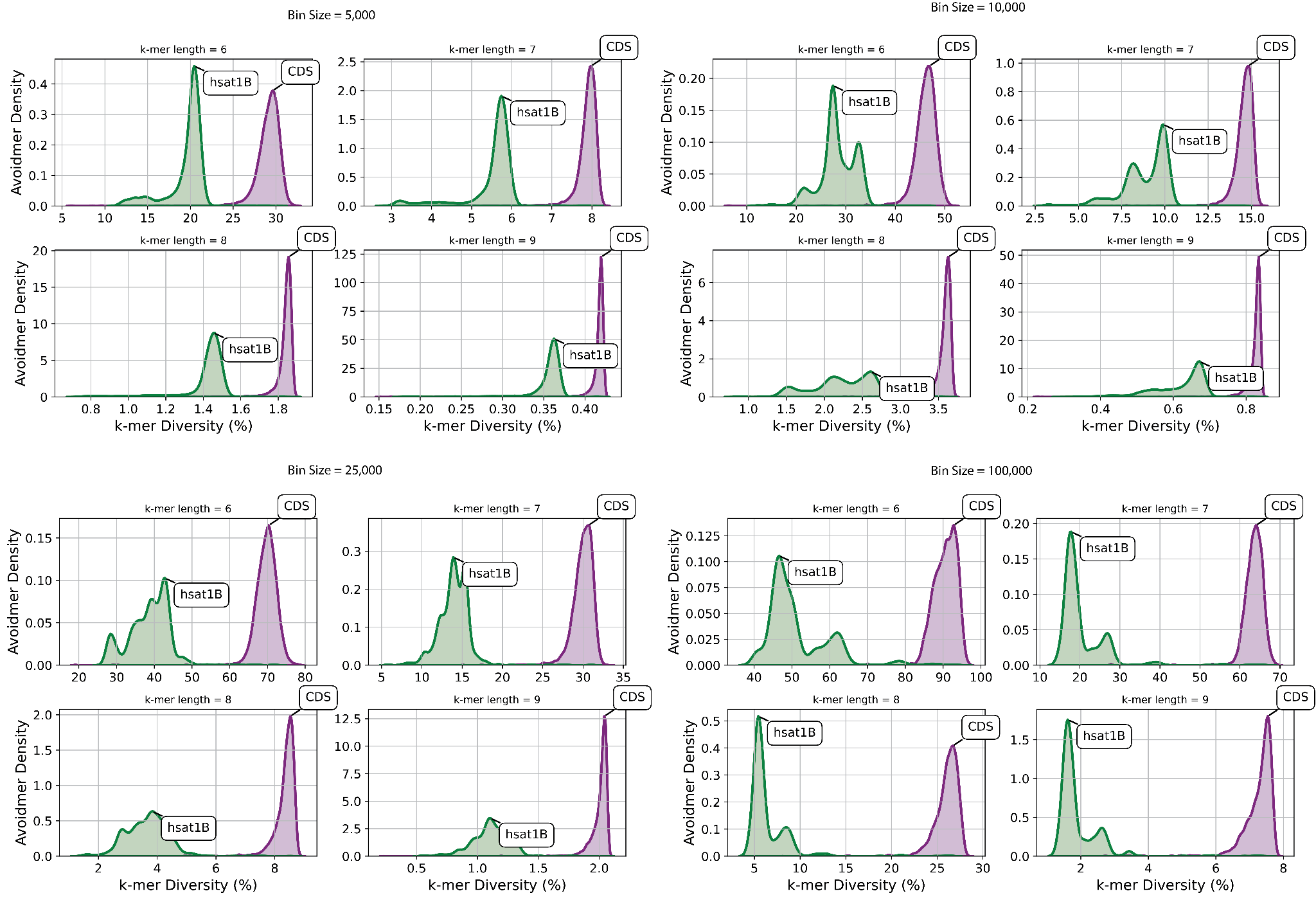


**Fig F. Kernel density estimation of k-mer diversity scores of 5kB, 10kB, 25kB, 100kB regions that belong either to hsat1B (green) or CDS (purple), contain a Zimin avoidmer of at least 50bp long, for k-mer lengths between six and nine base-pairs.**

**
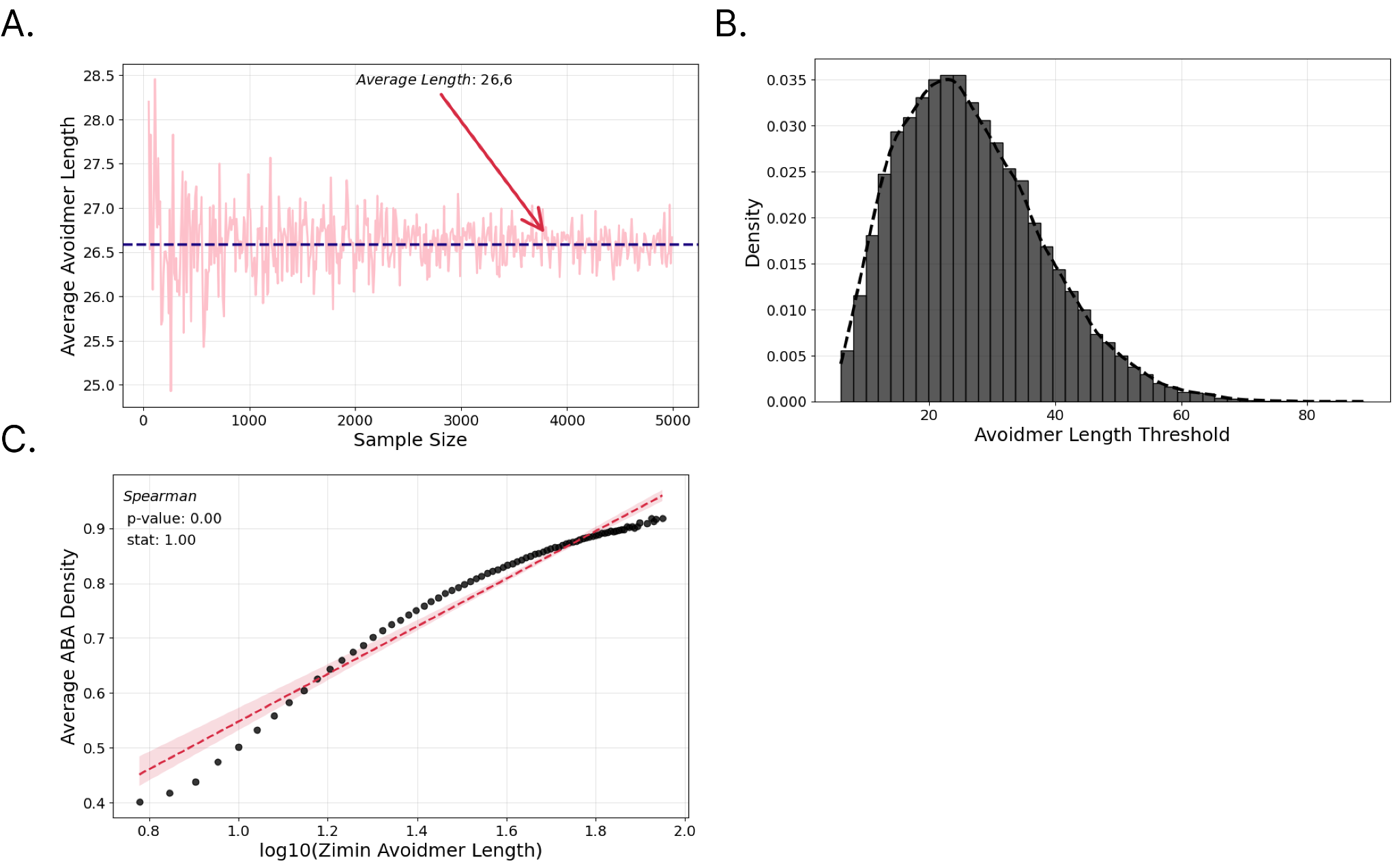
**

**Fig G. Examination of Zimin avoidmer length and composition distribution. A)** Average avoidmer length threshold approximation by increasing sample size, following the law of large numbers. Random sampling with replacement was used, with sample sizes ranging between 50 and 5,000, with 10 sample increments between iterations. **B)** Avoidmer length threshold distribution from random equidistributed simulations using the nucleotide alphabet. **C)** Average ABA Density is exponentially increased with Zimin avoidmer length.


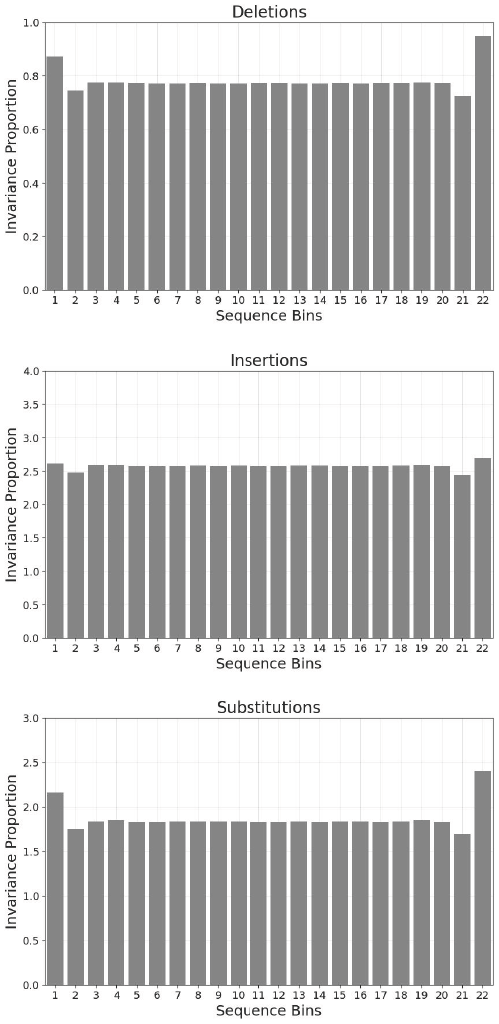


**Fig H. Mutational Invariance for Substitutions, 1bp Insertions, 1bp Deletions.** Each Zimin avoidmer was partitioned into twenty mutually exclusive bins and for each bin, the invariance potential for each of the mutations was estimated.


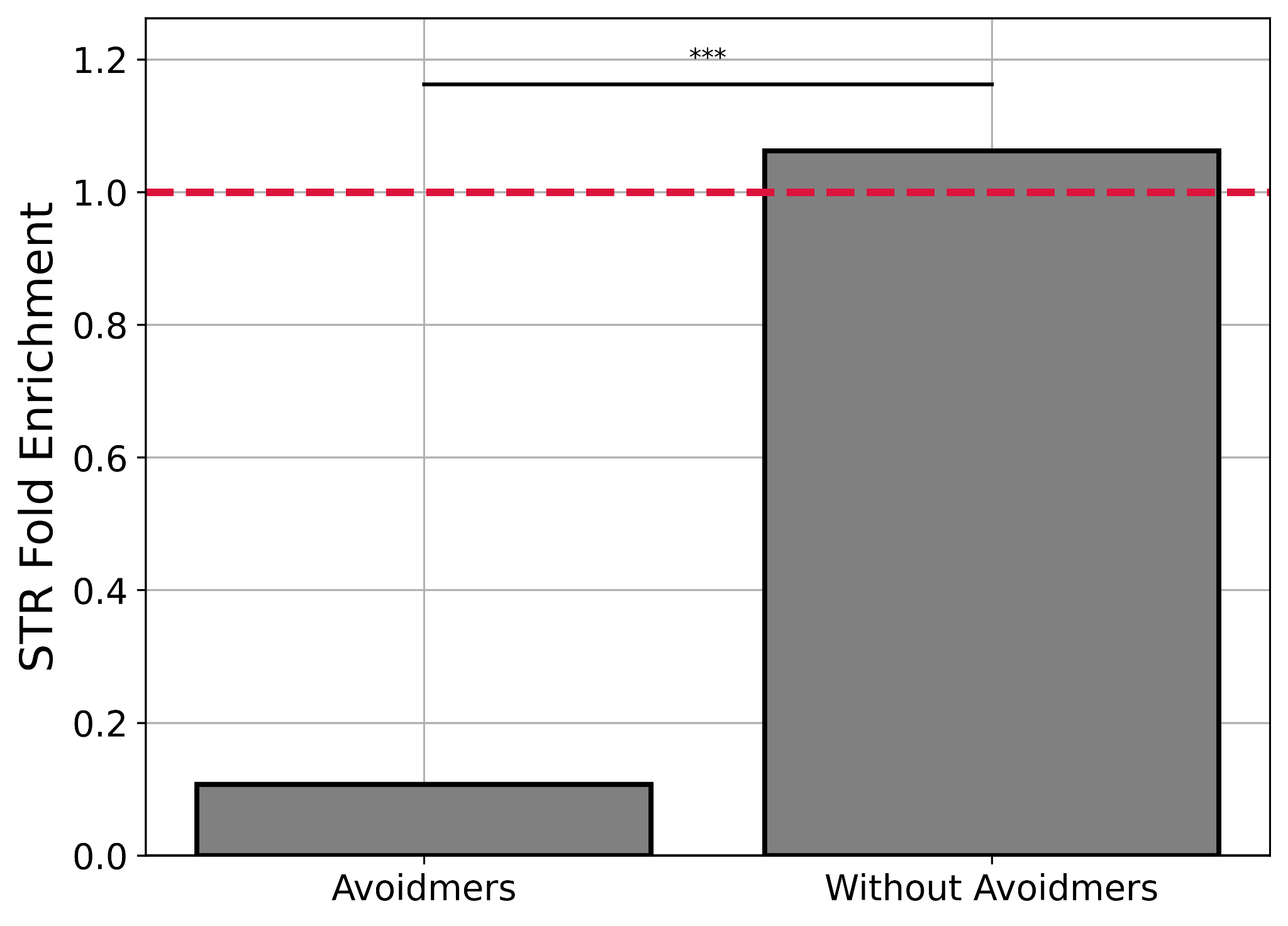


**Fig I. Comparison of STRs in Zimin avoiding regions and genomic regions that do not contain Zimin avoidmers in the human CHM13v2-T2T reference genome.** Enrichment represents the STR fold enrichment between the Zimin avoidmer regions and the regions that contain at least one Zimin word. Statistical significance is shown using Fisher's exact test (p-value<0.001).


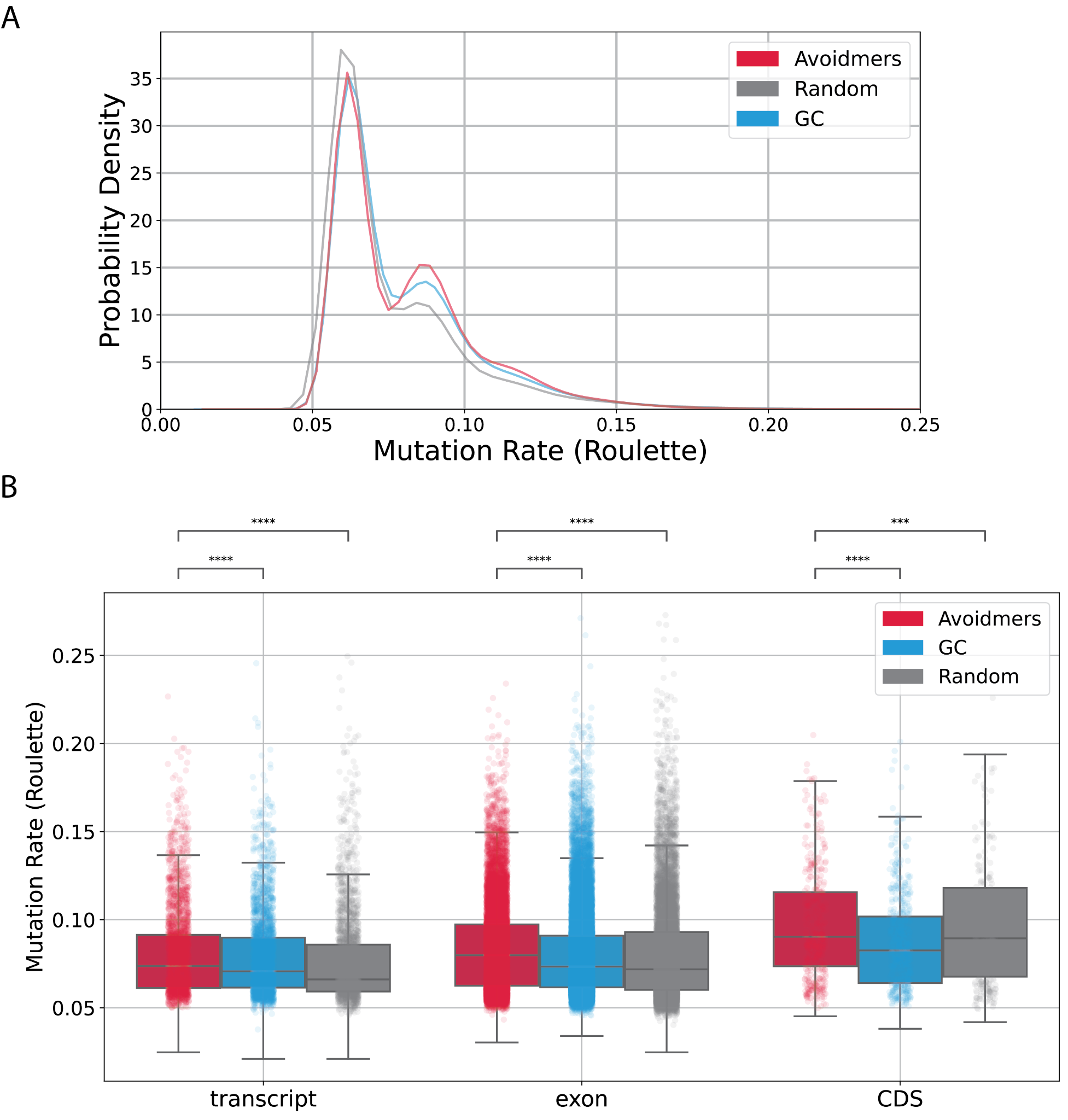
**Fig J. Predicted mutation rates of Zimin avoidmers compared to randomized and GC-Matched controls A.** Kernel density estimation of average predicted mutation rate by Roulette across three categories: Avoidmers, Randomized Controls, and GC-Matched Controls. **B.** Predicted mutation rates across Zimin avoidmers, Randomized Controls, and GC-Matched Controls across three genomic subcompartments, including transcripts, exons, and coding regions. Pairwise comparisons between each control group and their Zimin avoidmers have been conducted using two-tailed Mann-Whitney test, adjusted for multiple comparisons using Benjamini-Hochberg procedure. Adjusted p-values are displayed as * for p < 0.05, ** for p < 0.01, and *** for p < 0.001.


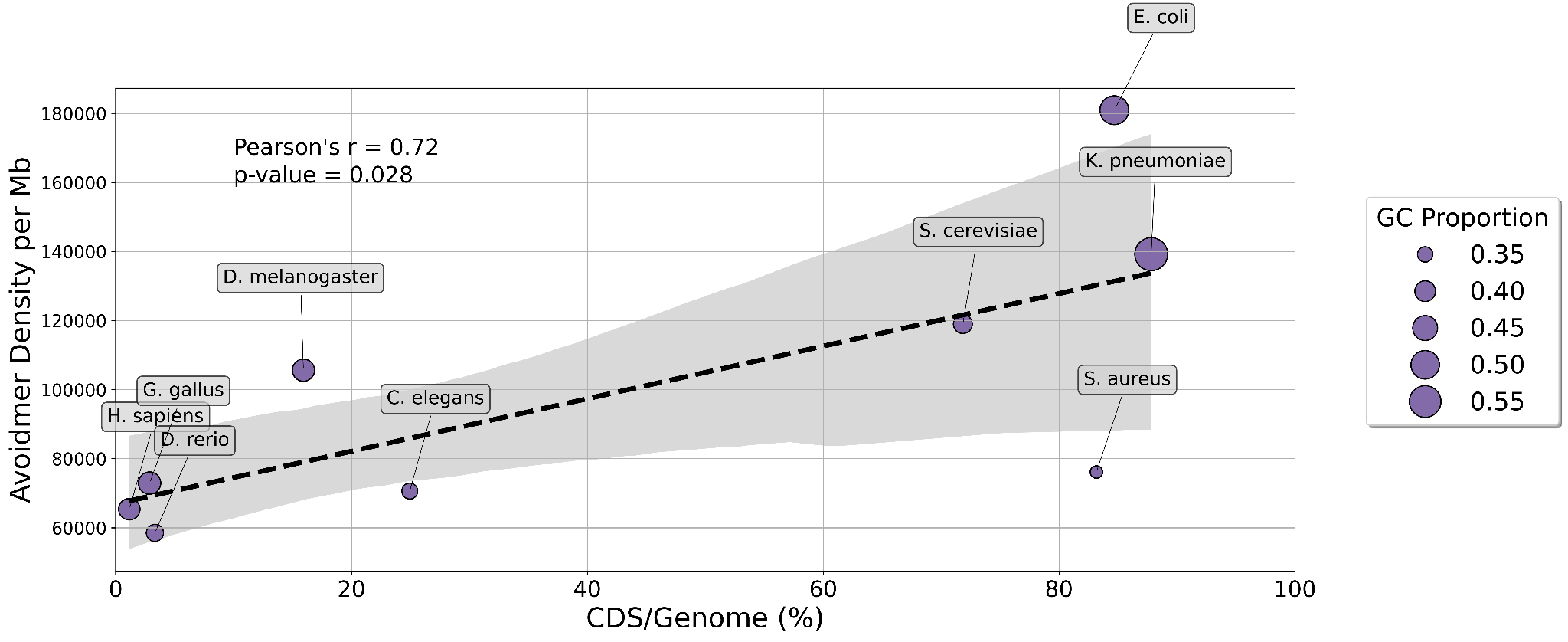


**Fig K. Linear regression bubbleplot of CDS to Genome ratio in relationship to Zimin avoidmer density per Mb. Additionally, the bubble size represents the genome-wide GC proportion for each organismal genome.**
