# Supplementary material for: Zimin patterns in genomes
Source: PLoS Comput Biol. 2026 Feb 9;22(2):e1013909. doi: 10.1371/journal.pcbi.1013909 (PMC12912701; doi:10.1371/journal.pcbi.1013909)
Supplement: S2 File — Table A: ABA Avoiding Expected Probabilities. Table B: ABACABA Avoiding Expected Probabilities. Table C: Longest Zimin avoidmers for k-mer lengths of 100bps or longer. Table D: Subsets of the most frequent Zimin avoidmers for selected compartments. The two color-highlighted sequences in Hsat1B differ by only 1 bp. Table E: Common Language Effect Size (CLES) of predicted mutation rates between each control group and Zimin avoidmers across three genomic subcompartments. Table F: p-values observed vs. expected probability model organisms, paired sample t-test. (DOCX) [file pcbi.1013909.s002.docx]

**Table A. ABA avoiding expected probabilities.**

| **K-mer Length** | **Total K-mers** | **ABA avoidmers** | **Expected Probability (%)** |
| --- | --- | --- | --- |
| 1 | 4 | 4 | 100.00 |
| 2 | 16 | 16 | 100.00 |
| 3 | 64 | 48 | 75.00 |
| 4 | 256 | 108 | 42.1875 |
| 5 | 1,024 | 168 | 16.40625 |
| 6 | 4,096 | 168 | 4.1015625 |
| 7 | 16,384 | 96 | 0.5859375 |
| 8 | 65,536 | 24 | 0.03662109375 |
| 9 | 262,144 | 0 | 0.00 |

**Table B. ABACABA avoiding expected probabilities.**

| **K-mer Length** | **Total k-mers** | **ABACABA avoidmers** | **Expected Probability (%)** |
| --- | --- | --- | --- |
| 6 | 4,096 | 4,096 | 100.00 |
| 7 | 16,384 | 16,320 | 99.609375 |
| 8 | 65,536 | 64,812 | 98.895263671875 |
| 9 | 262,144 | 256,536 | 97.8607177734375 |
| 10 | 1,048,576 | 1,011,948 | 96.50688171386719 |
| 11 | 4,194,304 | 3,978,168 | 94.84691619873047 |
| 12 | 16,777,216 | 15,586,224 | 92.9011344909668 |
| 13 | 67,108,864 | 60,859,008 | 90.6869888305664 |
| 14 | 268,435,456 | 236,831,208 | 88.22650015354156 |

**Table C. Longest Zimin avoidmers for k-mer lengths of 100bps or longer.**

| Chromosome | Start | End | Sequence | K-mer length |
| --- | --- | --- | --- | --- |
| chr1 | 227121938 | 227122040 | gagtagacttggcgtaattcttaacagtcatggaattgttgcaatggtgaatgggcattggctttaacttaaaagttaccagctgcattagcccctatcaag | 102 |
| chr1 | 227121943 | 227122046 | gacttggcgtaattcttaacagtcatggaattgttgcaatggtgaatgggcattggctttaacttaaaagttaccagctgcattagcccctatcaagagagtc | 103 |
| chr14 | 88541787 | 88541888 | aaggcaaagtcaacgttggctgaagccaggccccggtgatggttactcatgttcacataagcagcgttaggatccatggcctggacggtccagaggccgct | 101 |
| chr15 | 94141278 | 94141380 | ttcggtcaattggcaatcctgcactaggtggcaagtctatcaaaaatggattacctgggtaccatggcatttgcctgtagtcccagttactcaggaggctga | 102 |
| chr17 | 29407135 | 29407238 | gtcatggaaaaagccctggaattggaattgaatgatcctgagattatatcacagctctgcaacttactagctgtgtgacctaggtcagttggcttaacctttc | 103 |
| chr17 | 37605751 | 37605852 | cctgtaatcctagcactttggaaggccaaggtggatggataacttgaggtcaggggttcaagaccagcctggccaacatggcaaaaccctatctctactaa | 101 |
| chr19 | 8202184 | 8202285 | ctacggcaggttattcaattcatcgttgcttggatcaaaaacaaccgcctagaccaggcgtggtggctcaggtctgtaatcccagcattttgggaggctga | 101 |
| chr5 | 89164534 | 89164634 | aattcagtcgttatcaattcgggccaggtaccaaagtcactaacgtgtccaggttaactcctggaaggcagcaacattcttgcttttgatggtacgaata | 100 |
| chr7 | 57171035 | 57171139 | tcgttattgttaaggcataatgcagctggcgacttggagttgaagccattgctcatttaccattctgaaaaatcctagggcccttcagaattaagctacaccta | 104 |
| chr7 | 64843506 | 64843610 | taggtgtagcttaattctgaagggccctaggatttttcagaatggtaaatgagcaatggcttcaactccaagtcgccagctgcattatgccttaacaataacga | 104 |
| chr9 | 8763100 | 8763200 | aagctccggagttctaacgttgttagggaattaaggtccaatcaccaagcctagtccatgcttaggaatttccaaggtacttatacaggcaagtgaaagg | 100 |
| chrX | 118460314 | 118460414 | taaggattgcggtagctgccaatgccctgaggcagtggggccaacctaaagccaccaagtccagtcttttggataagtatgctaccggccgatgccaaga | 100 |
| chrX | 118526160 | 118526260 | tcttggcatcggccggtagcatacttatccaaaagactggacttggtggctttaggttggccccactgcctcagggcattggcagctaccgcaatcctta | 100 |

**Table D. Subsets of the most frequent Zimin avoidmers for selected compartments.** The two color-highlighted sequences in Hsat1B differ by only 1bp.

| **Sequence** | **Length** | **Occurrences** | **Compartment** |
| --- | --- | --- | --- |
| tcttcaccttgtgatccccttgccttggcctccaaatttgctgggattacaggcctgagccaagatccatatt | 73 | 4,212 | Hsat1B |
| tcttcaccttgtgatccccttgccttggcctccaaatttgctgggattacaggcctgagccaagatccgtatt | 73 | 277 | Hsat1B |
| tcttcaccttgtgatccccttgccttggcctccaaatttgctgggattacaggcctgagccaagatccatatt | 73 | 2,120 | Genic |
| ggaatcgcaaggaattgatgtgaacggaacggaatggaatggaatccaaagg | 52 | 2,074 | Genic |
| ggaatcgcaaggaattgatgtgaacggaacggaatggaatggaatccaaagg | 52 | 3,567 | Hsat3 |
| aatggactcctttggaatggtgtagtatgcaatgcaatcgactggcagggaatcaaaaggaat | 55 | 1,424 | Hsat3 |
| aatggtctagtatgcaatgcaatcgactggcagggaatcaaaaggaatgtaatcg | 55 | 1,311 | Hsat3 |
| gtagtatgcaatgcaatcgactggcagggaatcaaaaggaatgtaatggaat | 52 | 780 | Hsat3 |
| aatggactcgtttggaatggtctagtatgcaatgcaatcgactggcagggaatcaaaaggaat | 63 | 713 | Hsat3 |
| aatggtctagtatgcaatgcaatcgactggcagggaatcaaaaggaatgtaatcg | 55 | 1,311 | Hsat3 |
| gattccattgggttcaattcaatgatgattacattggattccgttctatg | 50 | 1,428 | Hsat2 |
| attcgcttgctttcgatgatgattccacttgagtccgttagaagattctattcaattacattcc | 64 | 910 | Hsat2 |
| ttccattcgcttgctttcgatgatgattccacttgagtccgttagaagattctattcaattacatt | 66 | 905 | Hsat2 |
| ttccacttgagtccgttagaagattctattcaattacattccatgacgattccg | 54 | 902 | Hsat2 |
| ccgttagaagattctattcaattacattccatgacgattccgttcgagtcca | 52 | 878 | Hsat2 |
| gatcatgttgttctttcggagtaacccctacttccagaataaagtgattaccaaggaat | 59 | 43 | CDS |
| cgggctatcactggcagttcggtgtcggagaacgcggccattgccatggctggaatagccaagctctttg | 70 | 31 | CDS |
| gttgctccaatacgtaaaaggcacttctgtagggctggcatgagtcagtcagttcaagacaacctgaagga | 71 | 17 | CDS |

**Table E. Common Language Effect Size (CLES) of predicted mutation rates between each control group and Zimin avoidmers across three genomic subcompartments.**

| **Compartment** | **Control Group** | **Common Language Effect Size (CLES)** |
| --- | --- | --- |
| Transcript | Random | 0.57 |
| Transcript | GC | 0.51 |
| Exon | Random | 0.57 |
| Exon | GC | 0.55 |
| CDS | Random | 0.51 |
| CDS | GC | 0.59 |

**Table F. p-values observed vs. expected probability model organisms, paired sample t-test.**

| Species | t-test statistic | Adjusted p-value |
| --- | --- | --- |
| *E. coli* | 2.3592 | 0.0400 |
| *K. pneumoniae* | 2.4110 | 0.0380 |
| *S. aureus* | 2.6985 | 0.0275 |
| *C. elegans* | 2.9204 | 0.0259 |
| *S. cerevisiae* | 2.9651 | 0.0259 |
| *D. melanogaster* | 3.0177 | 0.0259 |
| *G. gallus* | 3.0260 | 0.0259 |
| *D. rerio* | 3.3024 | 0.0259 |
