# Supplementary material for: Zimin patterns in genomes
Source: PLoS Comput Biol. 2026 Feb 9;22(2):e1013909. doi: 10.1371/journal.pcbi.1013909 (PMC12912701; doi:10.1371/journal.pcbi.1013909)
Supplement: Attachment — Submitted filename: Response_to_reviewers_Zimin_Avoidmers.docx [file pcbi.1013909.s004.docx]

**Reviewers' comments:**

Reviewer's Responses to Questions

**Comments to the Authors:
Please note here if the review is uploaded as an attachment.**

Reviewer #1: In this work, the authors aimed to investigate Zimin words (sequences with identical prefix and suffix) in genomic sequences, specifically exploring "Zimin avoidmers" - sequences that do not contain any Zimin patterns - across multiple organisms. The authors analyzed the Telomere-to-Telomere (T2T) complete human genome as well as other eight model organisms for Zimin words and Zimin avoidmers. In human genome, the authors found that all k-mers above 105 base-pairs contain Zimin words. The Zimin avoidmers have an inhomogeneous distribution in the human genome, and are most enriched in coding sequence (CDS) and human satellite 1 regions. Furthermore, the authors found Zimin avoidmers are associated with loci that have increased sequence diversity in the human genome, but they have a lower insertion and deletion mutation rates. Lastly, the authors identified Zimin avoidmers across model organism genomes and found that Zimin avoidmers are inhomogeneously distributed across model organismal genomes.

The study is well organized and the manuscript is well written. While to study the question of Zimin words in genomes is interesting, I have several concerns and suggestions relating to the biological implications and applications the study.

Major Comments:
1. While the study provides a novel computational approach to analyzing genomic sequences, the broader biological implications are not fully explored. The discussion section hints at potential connections to non-B DNA conformations but it would be better if the authors could expand the discussion on potential applications of Zimin words to synthetic biology and genome studies.

We would like to thank the reviewer for the comment. We agree that the broader biological implications of Zimin words, particularly in the context of synthetic biology and genome architecture, are important to expand upon. We have therefore added a new paragraph to the Discussion section outlining potential applications of Zimin avoidmers and patterns in synthetic design, genome engineering, and structural genomics.

We provide the updated text below:

“””

The ability of Zimin avoidmers to exclude repetitive patterns presents intriguing opportunities for synthetic biology and genome engineering. Synthetic DNA constructs often require the design of sequences with high sequence complexity, low repeat content and resistance to mutations, to ensure stability and minimize off-target effects [(Wang and Vasquez 2022; Renda, Hammerling, and Barrick 2014; Nuismer et al. 2021)](https://paperpile.com/c/k3UvUm/SCPA+HJ7w+QT5E). Given that Zimin avoidmers are complex sequences, and are depleted of repeats and indel mutations, Zimin avoidmers could be used to design mutation-resistant, low-repeat-content synthetic sequences, offering potential advantages for applications in biotechnology, synthetic biology, and genetic engineering. Additionally, their inherent resistance to insertions and deletions makes them attractive candidates for molecular barcoding, synthetic promoters, or as neutral scaffolds in genome editing platforms. In genome studies, Zimin words may serve as a theoretical tool to explore genomic complexity and repeat structure, and provide insights into the principles of genome architecture and evolution.

“””

2. The authors note a significant difference between the theoretical maximum Zimin word length and their observed maximum in all the model organisms. While they attribute this to the finite genome size and genome repetitiveness, a more in-depth mathematical and biological explanations of why this discrepancy occurs through mutation process and evolution would strengthen the manuscript.

We thank the reviewer for bringing up an important point. Our initial hypothesis was that Zimin avoidmer patterns, due to their pattern avoiding property, capture regions of increased sequence complexity. Eukaryotic genomes which are highly repetitive will be less likely to contain Zimin avoidmers than prokaryotes, which have less repeats. Thus, we expected lower repetitive element density to be linked with a higher density of Zimin avoidmer sequences. We have shown that repeat elements, such as tandem repeats are indeed depleted in Zimin avoidmers.

Following the reviewer’s suggestion, we provide additional evidence for this hypothesis. We have previously shown that organismal genomes, and in particular eukaryotic genomes, are highly enriched for repeat elements compared to simulated genomes that control for mono and dinucleotide content (https://www.biorxiv.org/content/10.1101/2024.08.08.607201v1.abstract).

We performed random permutations of the studied genomes, generating simulated versions for each genome while preserving the k-mer composition for k = 1 to 9. Our results show that the density of Zimin avoidmers increases in these simulated genomes when controlling for k-mer composition, and that this density decreases sharply as k-mer length increases. These findings support our initial hypothesis that Zimin avoidmers are positively correlated with genomic sequence diversity. The increase of Zimin avoidmers in simulated genomes is higher in eukaryotes than prokaryotes. Furthermore, less constrained simulated genomes (i.e. those simulated for controlling for lower k-mer length), tended to have longer Zimin avoidmer sequences (**Supplementary Figure 2c**).

Additionally, the theoretical maximum Zimin word length does not necessarily correspond to a tight upper bound. To investigate the tightness of the reported boundary, we used the longest Zimin avoiding sequences found in the human genome, and used a greedy algorithm to generate artificial very long Zimin avoiding sequences. We were able to construct a 388 base pairs long artificial Zimin avoidmer, 284 base pairs, higher than the largest avoidmer found in the human reference genome. We provide the Supplementary Figure 2 below:

**Supplementary Figure 2: Fold enrichment of avoidmers in simulated genomes of the human telomere-to-telomere reference genome**. **A)** Fold enrichment of avoidmer density of simulated shuffled genomes with respect to the reference genome, displayed in increasing order of preserved nucleotide composition starting from mononucleotides up to nine-nucleotides. The significance of the enrichment was evaluated using one-tailed binomial test, with background base rate being chosen as the the probability of a single base pair belonging to an avoidmer sequence of at least 50bp long, the number of positive events equal to the number of avoidmer base pairs in the shuffled genome, and the genome size as the total number of trials. **B)** Fold enrichment of avoidmer density of simulated shuffled genomes with respect to the human reference genome, as a function of the preserved nucleotide composition, for various minimum avoidmer thresholds. **C)** Maximum avoidmer length increase across shuffled genomes.

Therefore, we also provide more insights into Zimin avoidmers across evolution, by performing simulations in eight model organisms and examining the expected versus the observed number of Zimin avoidmers identified. The simulations ranged from controlling for 1bp to 7bp k-mer content in each organismal genome. We also now provide evidence that the Zimin avoidmer density is linked to the genomic complexity in a genome (**Figure 3C)**. Finally, we provide an association between the Zimin avoidmer density of an organismal genome and the proportion of the genome that is coding (**Supplementary Figure 10**). We provide the updated figure 5, Figure 3C and Supplementary Figure 10 and updated text below:

**Figure 5**: **Identification of avoidmers across model organisms. A)** Highest k-mer length at which avoidmers are observed in each chromosome in the human genome. **B)** Density of Zimin avoiding k-mers across model organisms. **C)** Expected and observed probability of k-mer being Zimin avoiding across model organisms. Colored lines represent the simulated genomes controlling but different k-mer lengths between one and seven base pairs. **D)** Observed probability of a k-mer being avoidmer across model organisms. **E)** Avoidmer percentage across genic and intergenic regions for each model organism. **F)** Zimin avoidmer percentage for increasing k-mer length for each of the under study model organisms. **G)** Zimin avoidmer density across genomic subcompartments for each model organism. Zimin avoidmer density was compared between genic and coding regions using independent t-tests, with multiple testing correction applied via the Benjamini–Hochberg procedure.

We also provide the updated text below:

“””
**Identification of Zimin words and Zimin avoidmers across model organism genomes**

Next, we examined how our findings translate in other organismal genomes. We selected a set of reference genomes from model organisms, spanning all three domains of life (**Table 1**), and estimated the k-mer length after which every k-mer contains a Zimin word. We observe that the highest Zimin avoidmer length is observed for *S. cerevisiae* for k-mer length of 115 bps, whereas the lowest is for *S. aureus* at 86 bps (**Figure 5a, Table 1**). We also find that the $Z_{3}$ avoidmer density of Zimin avoidmers of at least 50bp length, varies substantially between the examined organisms, ranging between 180,905.07 and 58,493.34 $Z_{3}$ avoidmers per mB in *E. coli* and *D. rerio* respectively (**Figure 5b**). We investigated the expected theoretical probability and observed probability of observing Zimin avoidmers in each of the model organisms. We find that the largest discrepancies between expected and observed probabilities are in eukaryotic organisms including *G. galus*, *D. rerio*, *D. melanogaster*, *S. cerevisiae* and *C. elegans*, whereas the smallest differences are observed in prokaryotes, namely *S. aureus*, *K. pneumoniae* and *E.coli* (**Figure 5c-d**). We shuffled each genome while controlling for nucleotide composition across k-mer lengths ranging from one to seven nucleotides. We observed that genomes shuffled with fewer constraints, specifically, those controlling for shorter k-mer lengths (lower k), converged more closely to the theoretical probability expected for Zimin avoidmers.(**Figure 5c**). This can be likely explained by the more repetitive and nucleotide imbalanced genomes of eukaryotes compared to prokaryotes.

“””

**Figure 3C:** Kernel density estimation of avoidmer density distribution, showing the complexity score per 1 kb genomic bins, based on Zimin avoidmers with a minimum length of 50 bp.

And the associated text for figure 3C:

“We also hypothesized that due to their inherent complexity, Zimin avoidmer sequences are abundant in parts of the genome with increased complexity. To test this hypothesis, we used data from Pirogov et al. [(Pirogov et al. 2021)](https://paperpile.com/c/k3UvUm/RubGW), in which the human genome is partitioned into mutually exclusive 1kB bins, each bin assigned a complexity value. We mapped Zimin avoidmer sequences of at least 50bp long, to a complexity bin. We report that the distribution of complexities of the mapped regions, is highly shifted towards the right, meaning that Zimin avoidmers have a tendency to occur in high complexity regions (**Figure 3c**). These findings support our hypothesis and suggest that Zimin avoidmers preferentially localize to structurally or compositionally complex regions of the genome.”

And Supplementary Figure 10:

**Supplementary Figure 10: Linear regression bubbleplot of CDS to Genome ratio in relationship to Zimin Avoidmer Density per Mb. Additionally, the bubble size represents the genome-wide GC proportion for each organismal genome.**

Minor Comments:
1. Figure 5 is blur and low resolution.

We apologize for this, we have now updated all the figures in the manuscript in high resolution. Additionally, we uploaded each figure in high resolution as a separate file.

2. Figure 5 legends, there are two B panels.

We apologize for this, we have now fixed this.

Reviewer #2: This manuscript investigates the Zimin pattern—where words have identical prefixes and suffixes—in the set of k-mers from the telomere-to-telomere (T2T) human reference genome, as well as reference genomes of eight other model organisms. The authors first explored the pattern of Zimin avoidmers, which are sequences that avoid the Zimin Zn pattern in the T2T genome. They concluded that for k-mer lengths exceeding 104 bp, every k-mer contains a Zimin word. Zimin avoidmers are enriched in coding regions and HSAT1B repetitive regions, and there is a negative correlation between k-mer diversity and the density of the Zimin pattern.

Next, the authors conducted simulations to examine the invariance of Zimin sequences to the introduction of indels and single nucleotide substitutions. They observed a strong enrichment or depletion of Zimin patterns around indels but not substitutions, likely due to nearby short tandem repeats. Finally, they compared Zimin avoidmers across the genomes of model organisms and note that the density of Zimin avoidmers is higher in prokaryotic genomes than in eukaryotic genomes.

Overall, the manuscript is well-written, and the analyses are robust. Below are some minor suggestions and general questions:

We would like to thank the reviewer for the overall positive assessment. We provide a point-by-point response below.

1. It would strengthen the manuscript to justify the examination of Zimin patterns in genomes by highlighting the repetitive nature of genomic sequences and presenting a specific hypothesis, rather than stating that “Zimin words have never been examined in bioinformatics.”

We would like to thank the reviewer. We have now explained the biological motivation emphasizing the need to understand repetitive patterns in genomes and provide the updated text below:

“A number of research papers have successfully applied theoretical mathematical concepts in genomics, such as the golden ratio, fractals and the Fibonnaci numbers among others [(Persaud and O’Leary 2015; Yamagishi and Shimabukuro 2008; Stanley et al. 1999)](https://paperpile.com/c/k3UvUm/BOe2+AHCq+Citw). Of high interest is the field of combinatorics as its theory and concepts can be applied in genomics in k-mer based investigations. A sesquipower or Zimin word is a sequence of characters over an alphabet with identical prefix and suffix, a concept that was proposed by A. I. Zimin over four decades ago [(Zimin 1984)](https://paperpile.com/c/k3UvUm/yrQw). Zimin words are unavoidable patterns, and all sufficiently long strings must contain Zimin words. The theoretical field of Zimin words has grown considerably since its inception and contains a sizable research literature [(Choffrut and Karhumäki 1997; Lothaire 2014; GöllerStefan 2019; Cooper and Rorabaugh 2014)](https://paperpile.com/c/k3UvUm/9vw1+ori1+VdB6+2IxH). Repetitive elements are prevalent in organismal genomes, with over half of the human genome composed of repetitive elements, including transposable elements, tandem repeats, and low-complexity regions (de Koning et al. 2011). Repetitive sequences play key roles in gene regulation, chromatin organization, and genome instability [(Wang and Vasquez 2023)](https://paperpile.com/c/k3UvUm/u3yJ). However, mathematical principles that govern these repetition and sequence structure remain incompletely understood. Zimin words are recursive, self-embedding patterns that are mathematically unavoidable in sufficiently long strings, and offer a theoretical lens through which the presence and organization of repetition in genomes can be studied. Despite their rich theoretical foundation in combinatorics on words, Zimin patterns have not been examined in biological sequences. We hypothesized that the distribution and avoidance of Zimin patterns in genomic sequences reflect fundamental constraints on genome structure, function, and evolution. Specifically, we propose that regions avoiding Zimin patterns, termed Zimin avoidmers, may serve as indicators of sequence complexity. By systematically characterizing Zimin words and avoidmers in the human genome and across model organisms, we aim to uncover new principles underlying genome organization and the limits of repeat avoidance.

”

2. Figure 3 analysis: Please provide a justification for focusing solely on 8-mers in the analysis presented in Figure 3.

We thank the reviewer for the comment and agree with the need to explain our choice. We have now performed the same analysis for k-mer lengths six to nine base-pairs to examine if there are differences in the findings and found that the results are highly consistent across k-mer lengths, indicating the validity of our findings We also provide the results for all k-mer lengths chosen below, in the results section describing them and in the updated figure.

“””Next, we binned the genome in non-overlapping 50 kilobase (kB) windows to examine how the k-mer diversity influences the number of avoidmers detected. In each bin we counted the number of unique k-mers detected for six to nine bps k-mer length. Then, we quantified the correlation between the avoidmer occurrences and distinct k-mer sequences detected in each bin. We observe that the Zimin avoidmers with the largest number of occurrences are primarily observed in bins in which there is large k-mer diversity (**Figure 3a; Supplementary Figure 4**). These results are also consistent in CDS compartments (**Figure 3b; Supplementary Figure 5**). However, we find that a small subset of Zimin avoidmers are found in bins that have low k-mer diversity and these originate from satellite repeat regions, such as hsat1B (**Figure 3b; Supplementary Figure 5**). The results were consistent for the different window sizes tests.

Next, to examine potential repetitive patterns in Zimin avoidmers, we investigated the density of subsequences that are an exact instance of $Z_{2}=aba$ in Zimin avoidmers [(Rorabaugh 2015)](https://paperpile.com/c/k3UvUm/ulIA). We observe that the density of ABA sequences in Zimin avoidmers follows a normal distribution; however with an elongated tail representing a small subset of Zimin avoidmers that have high density of ABA sequences (**Supplementary Figure 6a**). Interestingly, the average ABA density was anti-correlated with the number of distinct k-mers present in each Zimin avoidmer (**Supplementary Figure 6b**), indicating that, on average, Zimin avoidmers without ABA words have the highest k-mer diversity. In fact, on average, we anticipate the same effect in any k-mer. We conclude that Zimin avoidmers are associated with loci that have increased sequence diversity in the human genome.

We were interested to determine how long, on average, a uniformly randomly generated k-mer would be before it encounters an instance of $Z_{3}$. We simulated a simple random process which constructs a k-mer by choosing at random - with probability 0.25 - a nucleotide from the alphabet {a, g, c, t}. This random process continues until the resulting sequence encounters an instance of $Z_{3}$. We repeated this process 50,000 times and documented the resulting Zimin avoidmers for each of the random experiments. We estimated the average value of maximum length of randomly generated Zimin avoidmers being equal to 26.6bp (**Supplementary Figure 7a**). However, as quite a few outliers reach higher lengths (**Supplementary Figure 7b**). Finally, we examined the relationship of the lengths of the resulting sequences with the ABA density. The latter indicated a logarithmic relationship with the maximum lengths, suggesting that a small increase in the total length induces an exponential increase in the subsequences that are instances of $Z_{2}$ (**Supplementary Figure 7c**). Although this random process relies on basic assumptions that do not apply to regular genomes, such as nucleotide positional and local independence, it enables us to quantify the inherent difficulty of constructing arbitrarily long Zimin avoiding k-mers by relying purely on chance.

We also hypothesized that due to their inherent complexity, Zimin avoidmer sequences are abundant in parts of the genome with increased complexity. To test this hypothesis, we used data from Pirogov et al. [(Pirogov et al. 2021)](https://paperpile.com/c/k3UvUm/RubGW), in which the human genome is partitioned into mutually exclusive 1kB bins, each bin assigned a complexity value. We mapped Zimin avoidmer sequences of at least 50bp long, to a complexity bin. We report that the distribution of complexities of the mapped regions, is highly shifted towards the right, meaning that Zimin avoidmers have a tendency to occur in high complexity regions (**Figure 3c**). These findings support our hypothesis and suggest that Zimin avoidmers preferentially localize to structurally or compositionally complex regions of the genome.”””

**Figure 3: Characterization of the sequence diversity in avoidmers. A.** K-mer diversity scores of 50kB regions that contain a Zimin avoidmer of at least 50bp long, for k-mer lengths between six and nine base-pairs. **B-D.** K-mer diversity scores of 50kB regions that contain a Zimin avoidmer for k-mer lengths between six and nine base-pairs at two compartments: **B.** CDS, hsat1B classic satellite compartments. **C.** Kernel density estimation of avoidmer density distribution, showing the complexity score per 1 kb genomic bins, based on Zimin avoidmers with a minimum length of 50 bp.

**Supplementary Figure 5: Kernel density estimation of k-mer diversity scores of 5kB, 10kB, 25kB, 100kB regions that contain a Zimin avoidmer of at least 50bp long, for k-mer lengths between six and nine base-pairs.**

**Supplementary Figure 6: Kernel density estimation of k-mer diversity scores of 5kB, 10kB, 25kB, 100kB regions that belong either to hsat1B (green) or CDS (purple), contain a Zimin avoidmer of at least 50bp long, for k-mer lengths between six and nine base-pairs.**

3. In Figure 4C, it would be beneficial to include a comparison of short tandem repeat enrichment in regions that are not Zimin avoidmers.

We thank the reviewer for the comment. We did a comparison of STRs in Zimin avoiding regions and genomic regions that do not contain Zimin avoidmers. We report that STRs are significantly depleted in Zimin avoiding regions (two-tailed Fisher's Exact Test, p-value < 0.0001). We provide the results below:

“We performed a comparison of STRs in Zimin avoiding regions and genomic regions that do not contain Zimin avoidmers. We report that STRs are significantly depleted in Zimin avoiding regions (two-tailed Fisher's Exact Test, p-value < 0.001; **Supplementary Figure 9**).”

We also provide the updated methods section:

**Enrichment of STRs in regions at Zimin avoidmers**

We used the Zimin avoidmer coordinates to partition the human genome into two mutually disjoint groups: genomic regions that contain Zimin words and genomic regions that avoid them. Additionally, we used non-b GFA (Cer et al. 2013) to extract STRs in the CHM13v2-T2T human reference genome. We estimated the genome-wide STR density by dividing the total number of STR base pairs by the genome size. To avoid overcounting due to overlapping regions, we first merged overlapping STR intervals using the bedtools merge command. Subsequently, we used bedtools coverage to estimate the total density of regions containing Zimin words and those that avoid them. For each group, STR density was calculated by summing the number of base pairs overlapping with STRs and dividing by the total number of base pairs in the respective group. Fold enrichment was then computed as the ratio of the observed STR density within each group to the genome-wide STR density. Statistical significance was assessed using a two-sided Fisher’s exact test.

**Supplementary Figure 9: Comparison of STRs in Zimin avoiding regions and genomic regions that do not contain Zimin avoidmers in the human CHM13v2-T2T reference genome.** Enrichment represents the STR fold enrichment between the Zimin avoidmer regions and the regions that contain at least one Zimin word. Statistical significance is shown using Fisher's exact test (p-value<0.001).

4. Zimin Words in the Human Genome: What is the pattern of Zimin words (as opposed to Zimin avoidmers) in the human genome?

We thank the reviewer for the comment. Given a specific k-mer length, Zimin avoidmers above that threshold, partition the genome into two mutually disjoint regions. We provide below a link on UCSC browser with the Zimin avoidmer distribution of at least 50bp long across the human genome. Additionally, we provide two more distinct tracks, with the Zimin avoidmer density per 1kB and per 500bp throughout the human genome. The link for the UCSC browser tracks can be found in the provided GitHub page of our manuscript:

https://github.com/Georgakopoulos-Soares-lab/Avoidmers-DNA

or directly by accessing the following link:

[https://genome.ucsc.edu/cgi-bin/hgTracks?db=hub_3671779_hs1&lastVirtModeType=default[…]r1%3A1%2D610000&hgsid=2510082887_wZFzMPKwpFTHuq0psrdqPssw64ay](https://genome.ucsc.edu/cgi-bin/hgTracks?db=hub_3671779_hs1&lastVirtModeType=default&lastVirtModeExtraState=&virtModeType=default&virtMode=0&nonVirtPosition=&position=chr1%3A1%2D610000&hgsid=2510082887_wZFzMPKwpFTHuq0psrdqPssw64ay)

And provide in the text our GitHub repository:

**Code Availability**

The GitHub code and all the related material for this work is provided at: <https://github.com/Georgakopoulos-Soares-lab/Avoidmers-DNA/>

5. Reverse Complements of Zimin Sequences: Similarly, what is the pattern of the reverse complements of Zimin sequences? Exploring it may further elucidate the characteristics of Zimin patterns but not necessarily to address.

We would like to thank the reviewer for raising an interesting point. Zimin sequences are symmetric by definition. For every Zimin sequence its reverse complement is also a Zimin sequence and consequently for every Zimin avoidmer sequence its reverse complement is also a Zimin avoidmer. Therefore we decided to perform the analysis on a single strand. We have now clarified this by adding the following excerpt in the manuscript:

“Zimin sequences are symmetric by nature. As such, we have performed the avoidmer analysis on a single strand.”

Reviewer #3: The manuscript introduces the concept of Zimin avoidmers and examines their distribution and enrichment across the human reference genome and eight additional model organisms. Key findings include the inhomogeneous distribution of avoidmers, enrichment in coding regions and satellite sequences, and their association with sequence diversity, suggesting potential applications in understanding genomic sequence organization. While the manuscript presents an interesting mathematical concept applied to genomics, several significant concerns prevent me from recommending this submission for acceptance in its current form:

We thank the reviewer for their assessment of our work and helpful comments. We provide a point-by-point response below.

1. While the manuscript introduces an interesting theoretical concept’s application, it does not sufficiently explore its biological implications or potential applications, leaving the findings somewhat isolated from practical utility for computational biology. Without biological (either from functional or evolutionary) interpretation, this manuscript might be more suitable for journals focusing on computer science or natural language processing.

We thank the reviewer for the very important remark. We showed that eukaryotes and in particular complex multicellular organisms are less likely to contain Zimin avoidmers. Also, the ratio of CDS space to genome size is associated with the Zimin avoidmer density (**Supplementary Figure 10**). Also, we now show that genomic loci with highest genomic complexity, as previously defined by Pirogov et al. have the highest density of genomic avoidmers. Finally, we show that Zimin avoidmers are depleted of indels. This is early evidence that Zimin avoidmers and other pattern avoiding sequences can be useful in the characterization of genomic sequence complexity, in the design of synthetic genomes (please see Figure 5C) and in understanding the grammar and structure of genome language.

We provide the updated results:

“””We also hypothesized that due to their inherent complexity, Zimin avoidmer sequences are abundant in parts of the genome with increased complexity. To test this hypothesis, we used data from Pirogov et al. [(Pirogov et al. 2021)](https://paperpile.com/c/k3UvUm/RubGW), in which the human genome is partitioned into mutually exclusive 1kB bins, each bin assigned a complexity value. We mapped Zimin avoidmer sequences of at least 50bp long, to a complexity bin. We report that the distribution of complexities of the mapped regions, is highly shifted towards the right, meaning that Zimin avoidmers have a tendency to occur in high complexity regions (**Figure 3c**). These findings support our hypothesis and suggest that Zimin avoidmers preferentially localize to structurally or compositionally complex regions of the genome.”””

**Figure 3C.** Kernel density estimation of avoidmer density distribution, showing the complexity score per 1 kb genomic bins, based on Zimin avoidmers with a minimum length of 50 bp.

And the updated methods:

**Analysis of high complexity regions**

To assess if Zimin avoidmers are abundant in high complexity regions, we used data provided from Pirogov et al. [(Pirogov et al. 2021)](https://paperpile.com/c/vNm8Or/zECG). We used the provided bigWig file in which the GRCh38 is partitioned into mutually exclusive regions of 1kB long, each region assigned with a complexity score. We used liftOver to map the coordinates GRCh38 to CHM13v2-T2T genome and subsequently utilizing the command “bedtools intersect -wo f=1.0” we mapped the Zimin avoidmers to a unique complexity region. Finally, we plotted the distribution of the complexity scores using kdeplot as provided from Seaborn graphics library.

”””

We have added the following paragraph in the discussion to expand on their potential biological applications:
“
The ability of Zimin avoidmers to exclude repetitive patterns presents intriguing opportunities for synthetic biology and genome engineering. Synthetic DNA constructs often require the design of sequences with high sequence complexity, low repeat content and resistance to mutations, to ensure stability and minimize off-target effects [(Wang and Vasquez 2022; Renda, Hammerling, and Barrick 2014; Nuismer et al. 2021)](https://paperpile.com/c/k3UvUm/SCPA+HJ7w+QT5E). Given that Zimin avoidmers are complex sequences, and are depleted of repeats and indel mutations, Zimin avoidmers could be used to design mutation-resistant, low-repeat-content synthetic sequences, offering potential advantages for applications in biotechnology, synthetic biology, and genetic engineering. Additionally, their inherent resistance to insertions and deletions makes them attractive candidates for molecular barcoding, synthetic promoters, or as neutral scaffolds in genome editing platforms. In genome studies, Zimin words may serve as a theoretical tool to explore genomic complexity and repeat structure, and provide insights into the principles of genome architecture and evolution.

We have also revised the introduction:

“A number of research papers have successfully applied theoretical mathematical concepts in genomics, such as the golden ratio, fractals and the Fibonnaci numbers among others [(Persaud and O’Leary 2015; Yamagishi and Shimabukuro 2008; Stanley et al. 1999)](https://paperpile.com/c/k3UvUm/BOe2+AHCq+Citw). Of high interest is the field of combinatorics as its theory and concepts can be applied in genomics in k-mer based investigations. A sesquipower or Zimin word is a sequence of characters over an alphabet with identical prefix and suffix, a concept that was proposed by A. I. Zimin over four decades ago [(Zimin 1984)](https://paperpile.com/c/k3UvUm/yrQw). Zimin words are unavoidable patterns, and all sufficiently long strings must contain Zimin words. The theoretical field of Zimin words has grown considerably since its inception and contains a sizable research literature [(Choffrut and Karhumäki 1997; Lothaire 2014; GöllerStefan 2019; Cooper and Rorabaugh 2014)](https://paperpile.com/c/k3UvUm/9vw1+ori1+VdB6+2IxH). Repetitive elements are prevalent in organismal genomes, with over half of the human genome composed of repetitive elements, including transposable elements, tandem repeats, and low-complexity regions (de Koning et al. 2011). Repetitive sequences play key roles in gene regulation, chromatin organization, and genome instability [(Wang and Vasquez 2023)](https://paperpile.com/c/k3UvUm/u3yJ). However, mathematical principles that govern these repetition and sequence structure remain incompletely understood. Zimin words are recursive, self-embedding patterns that are mathematically unavoidable in sufficiently long strings, and offer a theoretical lens through which the presence and organization of repetition in genomes can be studied. Despite their rich theoretical foundation in combinatorics on words, Zimin patterns have not been examined in biological sequences. We hypothesized that the distribution and avoidance of Zimin patterns in genomic sequences reflect fundamental constraints on genome structure, function, and evolution. Specifically, we propose that regions avoiding Zimin patterns, termed Zimin avoidmers, may serve as indicators of sequence complexity. By systematically characterizing Zimin words and avoidmers in the human genome and across model organisms, we aim to uncover new principles underlying genome organization and the limits of repeat avoidance.

”

And Supplementary Figure 10:

**Supplementary Figure 10: Linear regression bubbleplot of CDS to Genome ratio in relationship to Zimin Avoidmer Density per Mb. Additionally, the bubble size represents the genome-wide GC proportion for each organismal genome.**

And updated our discussion with potential applications based on three advantages that Zimin avoidmers have: 1) they are depleted of repeats, 2) they have high sequence complexity, and 3) they are resistant to indels:

“The ability of Zimin avoidmers to exclude repetitive patterns presents intriguing opportunities for synthetic biology and genome engineering. Synthetic DNA constructs often require the design of sequences with high sequence complexity, low repeat content and resistance to mutations, to ensure stability and minimize off-target effects [(Wang and Vasquez 2022; Renda, Hammerling, and Barrick 2014; Nuismer et al. 2021)](https://paperpile.com/c/k3UvUm/SCPA+HJ7w+QT5E). Given that Zimin avoidmers are complex sequences, and are depleted of repeats and indel mutations, Zimin avoidmers could be used to design mutation-resistant, low-repeat-content synthetic sequences, offering potential advantages for applications in biotechnology, synthetic biology, and genetic engineering. Additionally, their inherent resistance to insertions and deletions makes them attractive candidates for molecular barcoding, synthetic promoters, or as neutral scaffolds in genome editing platforms. In genome studies, Zimin words may serve as a theoretical tool to explore genomic complexity and repeat structure, and provide insights into the principles of genome architecture and evolution.”

2. The study assumes that exact matches to Zimin words are meaningful, but this premise lacks biological plausibility. In genomic contexts, spontaneous mutations happen on the genome with certain rate, motifs or secondary structures like hairpin loops are typically error-tolerant and allow for mismatches or variations to some degree. Thus, focusing solely on exact matches to Zimin words is unrealistic and limits the biological relevance of the findings.

We thank the reviewer for the comment. Long Zimin avoidmers tend to be rare in a genome, whereas Zimin words are highly pervasive. The two sets are complementary. We do not allow mismatches when searching for Zimin words in genomes because Zimin words are defined by a strict recursive structure that relies on exact character placement. Unlike simple motifs or k-mers, where approximate matching can be defined through metrics such as Hamming or Levenshtein distance, even a single mismatch in a Zimin word disrupts the symmetry and self-similarity that define these patterns. Additionally, the vast majority of genomic space is Zimin words in our study. Allowing mismatches would therefore invalidate the formal properties of Zimin words and render the results uninterpretable within the existing mathematical framework. In this context, any deviation from the exact sequence breaks the recursive nature of the word and eliminates its classification as a Zimin pattern. As such, our analyses are restricted to exact matches in order to preserve the mathematical property of these patterns and the interpretability of the findings.

3. Statistical rigor is insufficient; there is no statistical significance reported throughout this manuscript. Claims regarding avoidmer distributions, enrichment in genomic compartments, and comparisons across organisms are made without appropriate statistical testing or measures of significance, undermining the reliability of the conclusions.

We thank the reviewer for the comment. We have now performed additional statistical tests which we provide below:

1) When comparing Zimin avoidmers with regions that do not contain any Zimin avoidmer, are significantly more GC rich (**Figure 1e;** Mann-Whitney U, p-value<0.0001).

2) We performed a paired sample t-test when comparing the expected Zimin avoiding probability with the observed Zimin avoiding probability in the human genome, and we report that the difference is significant with p-value < 0.01.

3) We performed one-tailed binomial test to assess the significance of the enrichment between the permuted genomes and the reference CHM13v2-T2T genome, and we report that the Zimin avoidmers were enriched in all permuted genomes (**Supplementary Figure 2a**; p-value < 0.001). We provide it also below:

4) We perfomed multiple comparisons across model organisms to evaluate the difference between the expected Zimin avoiding probability with the observed probability using a relative t-test, and adjusting for multiple hypothesis testing using Benjamini-Hochberg procedure. We report that all comparisons were significant (p-value < 0.05).

**Supplementary Table 5: p-values observed vs. expected probability model organisms, relative t-test.**

| Species | t-test statistic | Adjusted p-value |
| --- | --- | --- |
| *E. coli* | 2.3592 | 0.0400 |
| *K. pneumoniae* | 2.4110 | 0.0380 |
| *S. aureus* | 2.6985 | 0.0275 |
| *C. elegans* | 2.9204 | 0.0259 |
| *S. cerevisiae* | 2.9651 | 0.0259 |
| *D. melanogaster* | 3.0177 | 0.0259 |
| *G. gallus* | 3.0260 | 0.0259 |
| *D. rerio* | 3.3024 | 0.0259 |

5) In figure 5G, we now performed t-tests and provide the figure legend: “Zimin avoidmer density was compared between genic and coding regions using independent t-tests, with multiple testing correction applied via the Benjamini–Hochberg procedure.”

6) Performed Pearson correlation for the avoidmer density per MB versus the ratio of CDS/Genome content, which we provide below:

**Supplementary Figure 10: Linear regression bubbleplot of CDS to Genome ratio in relationship to Zimin Avoidmer Density per Mb. Additionally, the bubble size represents the genome-wide GC proportion for each organismal genome.**

10) We have incorporated Monte-Carlo simulations, for figure 2c:
For each genomic compartment we used bootstrap N=250 with replacement to construct confidence intervals for the avoidmer density. Error bars represent 95% confidence interval confidence.

11) We also provide below the results for the frequency of STRs in regions with Zimin avoidmers and in regions without them, in the human genome and the statistical analysis:

“We performed a comparison of STRs in Zimin avoiding regions and genomic regions that do not contain Zimin avoidmers. We report that STRs are significantly depleted in Zimin avoiding regions (two-tailed Fisher's Exact Test, p-value < 0.001; **Supplementary Figure 9**).”

**Supplementary Figure 9: Comparison of STRs in Zimin avoiding regions and genomic regions that do not contain Zimin avoidmers in the human CHM13v2-T2T reference genome.** Enrichment represents the STR fold enrichment between the Zimin avoidmer regions and the regions that contain at least one Zimin word. Statistical significance is shown using Fisher's exact test (p-value<0.001).

And we also provide the updated methods:

“**Enrichment of STRs in regions at Zimin avoidmers**

We used the Zimin avoidmer coordinates to partition the human genome into two mutually disjoint groups: genomic regions that contain Zimin words and genomic regions that avoid them. Additionally, we used non-b GFA (Cer et al. 2013) to extract STRs in the CHM13v2-T2T human reference genome. We estimated the genome-wide STR density by dividing the total number of STR base pairs by the genome size. To avoid overcounting due to overlapping regions, we first merged overlapping STR intervals using the bedtools merge command. Subsequently, we used bedtools coverage to estimate the total density of regions containing Zimin words and those that avoid them. For each group, STR density was calculated by summing the number of base pairs overlapping with STRs and dividing by the total number of base pairs in the respective group. Fold enrichment was then computed as the ratio of the observed STR density within each group to the genome-wide STR density. Statistical significance was assessed using a two-sided Fisher’s exact test.”

4. In the section "In the reference human genome every k-mer contains a Zimin word after 104 base-pairs," the statement "with a maximum Zimin avoidmer length of 104 bp emerging on chromosome 17" is inconsistent with Figure 1d, which indicates that chromosome 7 has the maximum avoidmer length.

We would like to thank the reviewer for the comment and apologize for the mistake. Chromosome 7 has the avoidmer of length 104bp. We have now corrected the phrase to:
“with maximum Zimin avoidmer length of 104 bp emerging on chromosome 7.”

5. In the section "In the reference human genome every k-mer contains a Zimin word after 104 base-pairs," the authors conclude that "Zimin avoidmers are significantly more GC-rich than the genome average." However, this conclusion should be drawn by comparing the GC content of Zimin avoidmers to that of k-mers containing Zimin words of the same specific length within the genome. Additionally, the authors should provide statistical evidence to support the significance of this difference.

We would like to thank the reviewer for the comment. Every part of the genome that is not a Zimin avoidmer contains Zimin patterns. Therefore, we compared all bps in the genome that have Zimin patterns to those that form Zimin avoidmers and compared their GC content. We report:

“We also performed genome simulations controlling for the k-mer composition from one up to nine base pairs. We observed that permuting the genome leads to an enrichment of Zimin avoidmer density across all examined k-mer lengths compared to the reference genome. In particular, we discover that Zimin avoidmers have a preference for balanced GC content that does not deviate in either high or low content (**Supplementary Figure 1**). The total Zimin avoidmer density decreases sharply with increasing simulated k-mer length (**Supplementary Figure 2a,b**, one-tailed binomial-test p-value < 0.001). Additionally, the maximum observed length of Zimin avoidmers is higher in all permuted genomes, with fixed mononucleotide composition displaying the maximum Zimin avoidmer length of 119 base pairs, accounting for approximately 14% total increase from the human reference genome (**Supplementary Figure 2c**). Thus, we conclude that the furthest we deviate from the reference genome, achieved using lower order permutations, the higher the Zimin avoidmer density and the longer the maximum observed Zimin avoidmer length. We conclude that every subsequence of the human genome, of at least 105 bps, contains a $Z_{3}$ Zimin pattern.”

We provide the updated methods below:

“””

**Controls for GC enrichment comparison**

We defined genomic regions in the human genome that do not contain any Zimin avoidmer sequence of at least 50bp long, using the BEDTools subtract command [(Quinlan and Hall 2010)](https://paperpile.com/c/k3UvUm/Prbi). We compared the GC-enrichment of these genomic regions to that of Zimin avoidmers of at least 50bp long, and used Mann-Whitney test to evaluate the statistical significance (**Figure 1e;** Mann-Whitney U, p-value<0.0001).

We partitioned the genome into mutually exclusive regions of 2,000kB. For each region, we calculated the GC proportion and the Zimin avoidmer fold enrichment by dividing the region's observed Zimin avoidmer density by the genome-wide density. We used a second-degree polynomial model to capture the relationship between GC content and Zimin avoidmer fold enrichment, employing the scikit-learn Python library. To determine the optimal region bin size and polynomial degree, we conducted a grid search with 10-fold cross-validation, using a polynomial feature transformer combined with a linear model. “””

And we provide the updated figure 1 (please see figure 1e, and updated supplementary figure 1, below):

**Figure 1**: **Identification of Zimin words and Zimin avoidmers in the human genome.** **A)** Number of Zimin avoiding k-mers as a function of k-mer length, for k-mers ranging between 30 and 100bps. **B-C)** Expected and observed probability of a k-mer being Zimin avoiding for **B.** n=2 and **C.** n=3. **D)** Highest k-mer length at which avoidmers are observed in each chromosome in the human genome. **E)** Comparison of GC enrichment in regions containing Zimin avoidmers versus regions lacking Zimin avoidmers of at least 50 bp in length. **F)** Enrichment of GC content in avoidmers over the human genome average, for avoidmers of increasing length.

**Supplementary Figure 1: Relationship of GC-Content and Avoidmer Density across the simulated genomes of human telomere-to-telomere reference genome.** For each simulated genome, the genome-wide avoidmer density was evaluated, and a second degree linear regression was used to model the relationship between the GC-content and avoidmer density.

6. In the section "Zimin avoidmers are inhomogeneously distributed across model organismal genomes," the authors claim that Zimin avoidmers are most enriched in genic and CDS regions of the genome. However, this claim is problematic because genic and CDS regions represent only a small fraction of the entire genome. The analysis does not account for the inherent bias introduced by sequence length, as longer sequences are inherently more likely to contain Zimin words due to their size. To make this claim more robust, the authors must normalize for sequence length to accurately assess enrichment and eliminate biases arising from differences in genomic region sizes.

We thank the reviewer for highlighting this issue. We have now clarified in the manuscript that these comparisons are done while taking into account the varying length of this regions and provide the updated text and updated figures below:

“For each examined organism we separated their genome in genic and intergenic regions and examined the percentage of Zimin avoidmers in each normalizing for region length. We find that across organisms, Zimin avoidmers have more occurrences in genic than intergenic regions (**Figure 5e-f**), results that are consistent with our observations for the human genome. When further separating the genomic compartments in genic, exonic and cds regions we find that the Zimin avoidmer density varies substantially both between sub-compartments and between the species (**Figure 5g**). Additionally, we report that Zimin avoidmer density increases with the CDS to genome ratio of an organism (**Supplementary Figure 10;** Pearson correlation: r=0.72, p-value < 0.05). We conclude that across most of the studied organisms, Zimin avoidmers are most enriched in genic and CDS regions of the genome, particularly in the case of organisms of eukaryotic origin.“

**Supplementary Figure 10: Linear regression bubbleplot of CDS to Genome ratio in relationship to Zimin Avoidmer Density per Mb. Additionally, the bubble size represents the genome-wide GC proportion for each organismal genome.**

**Figure 5**: **Identification of avoidmers across model organisms. A)** Highest k-mer length at which avoidmers are observed in each chromosome in the human genome. **B)** Density of Zimin avoiding k-mers across model organisms. **C)** Expected and observed probability of k-mer being Zimin avoiding across model organisms. Colored lines represent the simulated genomes controlling but different k-mer lengths between one and seven bps. **D)** Observed probability of a k-mer being avoidmer across model organisms. **E)** Avoidmer percentage across genic and intergenic regions for each model organism. **F)** Zimin avoidmer percentage for increasing k-mer length for each of the under study model organisms. **G)** Zimin avoidmer density across genomic subcompartments for each model organism. Zimin avoidmer density was compared between genic and coding regions using independent t-tests, with multiple testing correction applied via the Benjamini–Hochberg procedure.

7. In the section “In silico saturation and germline mutagenesis of Zimin avoidmers in the human genome,” the indels are constructed with a specific length of 1 (single-base insertion or deletion), which represents a highly idealized scenario. We suggest extending the analysis to include indels of varying lengths to better reflect real-world genomic mutational processes and evaluate whether the conclusions hold under more biologically realistic conditions.

We thank the reviewer for the remark. We examined the Zimin avoidmer invariance for random small deletions and small insertions. Here, we define invariance as the property of a Zimin avoidmer, to maintain Zimin avoidance, under a mutational transformation, such as small insertions and deletions. We performed a total of over 4 million tests, in which we selected two random positions in the Zimin avoidmer and examined if after their deletion the background sequence resulted in a Zimin avoidmer.

In particular, for each detected Zimin avoidmer, we deleted a random segment of the initial sequence, and examined if the Zimin avoidmer property remains invariant. We report that 87% of the total detected Zimin avoidmers were conserved under random deletions. Analogously, we performed the same random experiment for random insertions, by inserting a random nucleotide segment in the Zimin avoidmer sequence, and examined if the resulting sequence remains a Zimin avoidmer. We report that only the 13% of Zimin avoidmers were conserved under small insertions, with invariance being negatively correlated with insertion lengths. We conclude that Zimin avoidmers are more resilient under small deletions, rather than small insertions. This is also explained by the fact that the probability of Zimin avoidmers declines precipitously with increased k-mer length (Mann-Whitney-Wilcoxon test two-sided, p-value=0).

**Figure: Random simulation of Small Deletions and Small Insertions in Zimin avoidmers.** Simulated small deletions and small insertions of Zimin avoidmers in relationship to the deletion and insertion length, respectively.

8. Many of the parameter choices in the study require justification. It is important to assess whether the conclusions remain consistent when varying the bin window length of the genome. Exploring the validity of the findings under different parameter settings is necessary to ensure the robustness and generalizability of the results.

We thank the reviewer for their comment. We provide the following explorations to search across different parameters settings and report the consistency of our findings across them. These include:

1) Examining different k-mer lengths for detecting Zimin avoidmers.

We used 50 as the starting length of Zimin avoidmer sequences that we are interested in, as at lower lengths the majority of the sequences will be avoidmers. However, this parameter does not limit our search to higher length avoidmers, as our methodology includes extending every potential avoidmer sequence to its maximum length. To validate our methodology we repeated this analysis in the human genome using 60 as the starting length and we confirmed that the only sequences not identified through this new analysis were avoidmers of length between 50 and 60. Results for different k-mer lengths on finding Zimin avoidmers are shown in Figures 1, 2 and 5.

2) Examining k-mer diversity scores for k-mer lengths between six and nine base-pairs. We find that the results are largely consistent between k-mer lengths with the avoidmer density being concentrated at sites of high k-mer diversity as shown in Figure 3a-b; Supplementary Figure 4-5.

We provide the updated text and supplementary figure for the different k-mer lengths below:

“””Next, we binned the genome in non-overlapping 50 kilobase (kB) windows to examine how the k-mer diversity influences the number of avoidmers detected. In each bin we counted the number of unique k-mers detected for six to nine bps k-mer length. Then, we quantified the correlation between the avoidmer occurrences and distinct k-mer sequences detected in each bin. We observe that the Zimin avoidmers with the largest number of occurrences are primarily observed in bins in which there is large k-mer diversity (**Figure 3a; Supplementary Figure 4**). These results are also consistent in CDS compartments (**Figure 3b; Supplementary Figure 5**). However, we find that a small subset of Zimin avoidmers are found in bins that have low k-mer diversity and these originate from satellite repeat regions, such as hsat1B (**Figure 3b; Supplementary Figure 5**). The results were consistent for the different window sizes tests.”””

**Supplementary Figure 4: ABA sub-sequence density in avoidmers and its relationship with canonical k-mer diversity. A.** ABA sub-sequence density in avoidmer sequences. **B.** ABA density in avoidmers as a function of average canonical k-mer diversity for various k-mer lengths.

3) We have now also performed genome simulations, controlling for k-mer content for k-mer lengths of 1bp to 9bps. We showed that for low k-mer lengths we identify significantly more Zimin avoidmers and as we increased the k-mer length in the simulations, the number of Zimin avoidmers detected approximates those identified in the human genome. This indicates the lower number of Zimin avoidmers relative to the theoretical threshold is due to the highly repetitive nature of the human genome.

We provide the updated methods and results below:

**Performance of Genome Simulations**

We used the ushuffle package to perform genome-wide simulations while controlling for nucleotide composition based on the frequency of the k-mer content [(Jiang et al. 2008)](https://paperpile.com/c/k3UvUm/iXV9). For each studied organismal genome we generated nine simulated genomes, each time controlling for one up to nine base pair k-mer frequencies. From the resulting simulated genomes, we extracted the Zimin avoidmers of length at least 50bp long to estimate their genome-wide frequency. Compared to every simulated genome with a fixed k-mer composition, the fold enrichment was calculated as the ratio between the total density of Zimin avoidmer occurrences in the original (unshuffled) genome and their corresponding density in the simulated genome,, i.e.

$$FE=\frac{\#ZA(k)}{\#ZA},$$

where with *#ZA* we denote the total number of Zimin Avoidmer base pairs in the reference genome, and with *#ZA(k)* the total number of Zimin Avoidmer base pairs in the simulated genome with fixed k-mer length composition.

For each genome, the Zimin avoidmer density was estimated by calculating the total number of Zimin avoidmer base pairs divided by the total genome size. To ensure that we do not overcount the same base pairs twice, we merged the overlapping Zimin avoidmers, using bedtools merge command [(Quinlan and Hall 2010)](https://paperpile.com/c/k3UvUm/Prbi). Additionally, to evaluate the significance of the observed enrichment between the permuted and the reference genomes, we used one-tailed binomial test, as provided from python SciPy package, using as base probability, the Zimin avoidmer density of the reference genome, the genome size as the sample space size, and the observed Zimin avoidmer base pairs in the simulated genome as the total number of trials.

Updated the results section:

“””We also performed genome simulations controlling for the k-mer composition from one up to nine base pairs. We observed that permuting the genome leads to an enrichment of Zimin avoidmer density across all examined k-mer lengths compared to the reference genome. In particular, we discover that Zimin avoidmers have a preference for balanced GC content that does not deviate in either high or low content (**Supplementary Figure 1**). The total Zimin avoidmer density decreases sharply with increasing simulated k-mer length (**Supplementary Figure 2a,b**, one-tailed binomial-test p-value < 0.001). Additionally, the maximum observed length of Zimin avoidmers is higher in all permuted genomes, with fixed mononucleotide composition displaying the maximum Zimin avoidmer length of 119 base pairs, accounting for approximately 14% total increase from the human reference genome (**Supplementary Figure 2c**). Thus, we conclude that the furthest we deviate from the reference genome, achieved using lower order permutations, the higher the Zimin avoidmer density and the longer the maximum observed Zimin avoidmer length. We conclude that every subsequence of the human genome, of at least 105 bps, contains a $Z_{3}$ Zimin pattern.”””

And the updated supplementary figures:

**Supplementary Figure 1: Relationship of GC-Content and Avoidmer Density across the simulated genomes of human telomere-to-telomere reference genome.** For each simulated genome, the genome-wide avoidmer density was evaluated, and a second degree linear regression was used to model the relationship between the GC-content and avoidmer density.

**Supplementary Figure 2: Fold enrichment of avoidmers in simulated genomes of the human telomere-to-telomere reference genome**. **A)** Fold enrichment of avoidmer density of simulated shuffled genomes with respect to the reference genome, displayed in increasing order of preserved nucleotide composition starting from mononucleotides up to nine-nucleotides. The significance of the enrichment was evaluated using one-tailed binomial test, with background base rate being chosen as the the probability of a single base pair belonging to an avoidmer sequence of at least 50bp long, the number of positive events equal to the number of avoidmer base pairs in the shuffled genome, and the genome size as the total number of iterations. **B)** Fold enrichment of avoidmer density of simulated shuffled genomes with respect to the human reference genome, as a function of the preserved nucleotide composition, for various minimum avoidmer thresholds. **C)** Maximum avoidmer length increase across shuffled genomes.

4) We performed the analysis for both different window sizes, (100kB, 50kB, 25kB, 10kB, 5kB) and for k-mer lengths of six to nine base-pairs, and report highly consistent resutls (Supplementary Figures 3-4). We provide the updated results below:

Updated results section:
“””Next, we binned the genome in non-overlapping 50 kilobase (kB) windows to examine how the k-mer diversity influences the number of avoidmers detected. In each bin we counted the number of unique k-mers detected for six to nine bps k-mer length. Then, we quantified the correlation between the avoidmer occurrences and distinct k-mer sequences detected in each bin. We observe that the Zimin avoidmers with the largest number of occurrences are primarily observed in bins in which there is large k-mer diversity (**Figure 3a; Supplementary Figure 4**). These results are also consistent in CDS compartments (**Figure 3b; Supplementary Figure 5**). However, we find that a small subset of Zimin avoidmers are found in bins that have low k-mer diversity and these originate from satellite repeat regions, such as hsat1B (**Figure 3b; Supplementary Figure 5**). The results were consistent for the different window sizes tests.“””

Updated methods section:

“””

**Quantification of k-mer diversity across the human genome**

A k-mer w is defined as any sequence of k letters drawn from the nucleotide alphabet $A=\{a, g, c, t\}$. The length of the k-mer w is denoted as $|w|=k$. Given a k-mer w, we define the subsequence $w[i:i+l]$ of w, as the $l$*-*mer that starts at position $0\leq i\leq|w|-l$ and ends at position $i+l-1$(inclusive) with $1\leq l\leq|w|$. Given a k-mer *w*, we define the canonical k-mer *C(w)*, as the k-mer or its reverse-complement that is minimum in respect to the lexicographic order. For a given genomic region *E* and an integer number *k*, we define the *k-*diversity of *E,* denoted by *div(E, k),* as the ratio of the number of unique canonical k-mers within *E* to the total number of possible canonical k-mers *N(k)*. Thus,

$$div(E, k)=\frac{|\{w[i:i+k]: 0\leq i\leq|E|-k+1\}|}{N(k)},$$

where $N(k)=\frac{4^{k}-4^{0.5k}}{2}+4^{0.5k}, k\mathbb{\in N}$, is the total number of unique canonical k-mers.

To investigate the relationship of avoidmers to high complexity genomic areas, we partitioned each chromosome of the *Homo sapiens* T2T assembly into non-overlapping, consecutive 5kB, 10kB, 25kB, 50kB, 100kB window bins, respectively. For each of the bins, we evaluated the associated k-mer diversity as defined above for k=6, 7, 8, and 9 base pairs. Subsequently, we calculated the number of Zimin avoidmers in each genomic bin. Then we compared the association between the k-mer diversity and the total number of occurrences of Zimin avoidmers.

“””

And updated supplementary figures below:

**Supplementary Figure 5: Kernel density estimation of k-mer diversity scores of 5kB, 10kB, 25kB, 100kB regions that contain a Zimin avoidmer of at least 50bp long, for k-mer lengths between six and nine base-pairs.**

**Supplementary Figure 6: Kernel density estimation of k-mer diversity scores of 5kB, 10kB, 25kB, 100kB regions that belong either to hsat1B (green) or CDS (purple), contain a Zimin avoidmer of at least 50bp long, for k-mer lengths between six and nine base-pairs.**

9. The introns, exons, and CDS regions of a gene are not consecutive but rather scattered as separate segments across the genome. It is unclear how Zimin avoidmers are calculated for these fragmented regions. Does the analysis involve concatenating these segments into a single sequence, or are avoidmers identified separately within each segment? This methodological detail is crucial and should be explicitly described to clarify the approach used for Zimin avoidmer identification in these regions.

We would like to thank the reviewer for their comment. Zimin avoidmers were identified in the genome and their coordinates were identified. We then examined if these coordinates overlapped with different genomic subcompartments. The overlap had to be at least one base pair between the Zimin avoidmer coordinates and the genomic compartment avoidmer. This allowed us to estimate per base-pair the density of each Zimin avoidmer in each compartment. However, when we annotated Zimin avoidmers, these were annotated as belonging to a genomic compartment if at least 80% of the Zimin avoidmer sequence was found at that compartment. We now provide the updated text in the methods section regarding that:

“To assess the genomic context of Zimin avoidmers, we identified their coordinates in the genome and quantified overlaps with various genomic subcompartments, requiring a minimum of one base-pair overlap between them. The density of Zimin avoidmers in genic, exonic, 5' UTR, 3' UTR, and CDS regions as well as in centromeric and pericentromeric repeat regions was calculated using publicly available GTF and GFF coordinate files. Additionally, for the prokaryotic genomes we used agat [(NBISweden/AGAT: AGAT-v1.4.1)](https://paperpile.com/c/vNm8Or/wWyEc) to fill up the missing exon annotations. For each annotated subcompartment, overlapping compartments of the same type were merged and expanded into non-overlapping, mutually disjoint sets of coordinates. These coordinates were then used to estimate the density per Mb by calculating the total number of bps of avoidmers divided by the total compartment length of the previously merged genomic regions. Furthermore, to estimate the total percentage of genic and intergenic avoidmers, a Zimin avoidmer was classified as genic, if it had at least 80% overlap with a genic region.”

10. Figures (such as Figure 2) are not aligned between the subplots, and the writing should also be improved.

We thank the reviewer. We have now carefully gone through the figures and made sure everything aligns and we also worked towards improving the quality of the text throughout the manuscript.
