# Supplementary material for: Zimin patterns in genomes
Source: PLoS Comput Biol. 2026 Feb 9;22(2):e1013909. doi: 10.1371/journal.pcbi.1013909 (PMC12912701; doi:10.1371/journal.pcbi.1013909)
Supplement: Attachment — Submitted filename: RTR 2 Zimin.docx [file pcbi.1013909.s005.docx]

**Reviewers' comments:**

Reviewer's Responses to Questions

**Comments to the Authors:
Please note here if the review is uploaded as an attachment.**

Reviewer #1: Review comments (Remarks to the authors)
Thanks to the authors for their detailed and comprehensive additional analysis to address my comments. Most of my comments have been addressed in this revision. I just have a few more comments based on the authors’ response.
Major:
1.The authors addressed my second comment on mathematical and biological explanations of the Zimin avoidmer lengths. I like the author’s analysis on random permutations of the studied genomes for the mathematical explanations. For biological explanations, in additional to the genome complexity, I’d like to see if the Zimin avoidmer pattern is associated with mutation rates (there are multiple mutation rate models, for example, the authors could find the mutation rates in human genome here: <https://www.nature.com/articles/s41588-023-01562-0>) or the genome constraint in human genome (for example, the gnocchi score, <https://www.nature.com/articles/s41586-023-06045-0>). Such analysis would be interesting to broader readers, especially in the human genetics field.
Minor:
1.Figure 5A and 5B captions are mis-matched.

We thank the reviewer for the overall positive assessment. We provie the updated text below:

“””

We conducted an analysis to investigate the association between mutation rate in the human genome and Zimin avoidmer patterns. At first, we used hypermutable sites predicted by Roulette [(Seplyarskiy et al. 2023)](https://paperpile.com/c/7CV8MA/PfEh) and examined if these loci tend to co-localize within Zimin avoidmer defined genomic regions. We report that out of 2,020 hypermutable sites predicted by Roulette, the 6.25% (n=161) lie within Zimin avoidmer loci, which constitutes a 1.3-fold enrichment from the genome-wide background rate (p-value < 0.001, Fisher’s exact test).

We examined if the Zimin avoidmer regions exhibit higher mutation rates than expected, we constructed two control sets: randomized shuffled controls (Random group), and GC-matched controls (GC group). We conducted an investigation of the distribution of predicted mutation rate by Roulette density within Zimin avoidmers and each aforementioned control group. We report that all groups exhibit a bimodal distribution with Zimin avoidmers, followed by the GC-matched control group exhibiting a slightly higher concentration of higher mutation rate (**Supplementary Figure 9A**). Additionally, we investigated if the mutation rates differ across the genomic subcompartments. We report that, while Zimin avoidmers exhibit significantly higher mutation rates than the two control groups across genic, exonic and coding regions (two-tailed Mann-Whitney U, adjusted for multiple comparisons using Benjamini-Hochberg procedure) (**Supplementary Figure 9B**), the common language effect size differences between the Zimin avoidmers and the two constructed control groups suggest that the observed differences are negligible (**Supplementary Table 5**).

**Supplementary Figure 9: Predicted mutation rates of Zimin avoidmers compared to randomized and GC-Matched controls A.** Kernel density estimation of average predicted mutation rate by Roulette across three categories: Avoidmers, Randomized Controls, and GC-Matched Controls. **B.** Predicted mutation rates across Zimin avoidmers, Randomized Controls, and GC-Matched Controls across three genomic subcompartments, including transcripts, exons, and coding regions. Pairwise comparisons between each control group and their Zimin avoidmers have been conducted using two-tailed Mann-Whitney test, adjusted for multiple comparisons using Benjamini-Hochberg procedure. Adjusted p-values are displayed as * for p < 0.05, ** for p < 0.01, and *** for p < 0.001.

**Supplementary Table 5: Common Language Effect Size (CLES) of predicted mutation rates between each control group and Zimin avoidmers across three genomic subcompartments.**

| **Compartment** | **Control Group** | **Common Language Effect Size (CLES)** |
| --- | --- | --- |
| Transcript | Random | 0.57 |
| Transcript | GC | 0.51 |
| Exon | Random | 0.57 |
| Exon | GC | 0.55 |
| CDS | Random | 0.51 |
| CDS | GC | 0.59 |

Additionally, we have included a new Methods section which describes the methodology:

“””

**Estimation of Predicted Mutation Rates within Zimin avoidmers and matched controls**

For each Zimin avoidmer we created matched randomized controls using BEDTools shuffle command. To assess the influence of GC-bias of Zimin avoidmers, we constructed an additional GC-content matched control group using an in-house python script. We used predicted mutation rates predicted by Roulette for each chromosome using data from [(Seplyarskiy et al. 2023)](https://paperpile.com/c/7CV8MA/PfEh). We downloaded hypermutable sites from <http://genetics.bwh.harvard.edu/downloads/Vova/Roulette/hypermutable/>. Additionally, we downloaded all the predicted mutation rates from: <http://genetics.bwh.harvard.edu/downloads/Vova/Roulette/>. Each file was processed and the predicted mutation rates across the mutation spectrum were averaged into a single mutation rate per loci. Subsequently, for each element across the three groups: Zimin avoidmers, randomized controls, GC-matched controls, we calculate a single average mutation rate, using the bedtools map function. The statistical comparisons were conducted using two-tailed Mann-Whitney test from the SciPy Python package. The visualizations were performed using seaborn Python library. The jitter plot was randomly sampled from each group corresponding to 1% of the total population for each genomic subcompartment.

“””

**Have the authors made all data and (if applicable) computational code underlying the findings in their manuscript fully available?**
The [PLOS Data policy](https://nam10.safelinks.protection.outlook.com/?url=https%3A%2F%2Ftrack.editorialmanager.com%2FCL0%2Fhttps%3A%252F%252Fjournals.plos.org%252Fploscompbiol%252Fs%252Fmaterials-and-software-sharing%2F1%2F010f019961039db7-2517fabd-5902-4737-9a78-f203d63d7f03-000000%2Fl90lrnTKIF8zOSp9isxP7CGkk5GZrZnEOVumb3BcT-M%3D229&data=05%7C02%7Cizg5139%40psu.edu%7Cf850195f53124dcf5bc808ddf7539552%7C7cf48d453ddb4389a9c1c115526eb52e%7C0%7C0%7C638938660552642620%7CUnknown%7CTWFpbGZsb3d8eyJFbXB0eU1hcGkiOnRydWUsIlYiOiIwLjAuMDAwMCIsIlAiOiJXaW4zMiIsIkFOIjoiTWFpbCIsIldUIjoyfQ%3D%3D%7C0%7C%7C%7C&sdata=w8wnm9eON%2F1O2%2B7CUhqHXoNZyvY19rn5llzhkdk5mEs%3D&reserved=0) requires authors to make all data and code underlying the findings described in their manuscript fully available without restriction, with rare exception (please refer to the Data Availability Statement in the manuscript PDF file). The data and code should be provided as part of the manuscript or its supporting information, or deposited to a public repository. For example, in addition to summary statistics, the data points behind means, medians and variance measures should be available. If there are restrictions on publicly sharing data or code —e.g. participant privacy or use of data from a third party—those must be specified.

We have complied with computational code and data being all available, have released our code and data in Zendo and also provide summary statistics. The updated text is found below:

“””

**Data Availability**
All the source data and the scripts in this study can be found in the Zenodo repository (<https://zenodo.org/records/17774048>). The eukaryotic model organisms were downloaded directly from UCSC genome browser [(Perez et al. 2025)](https://paperpile.com/c/k3UvUm/QAee). The bacterial reference genomes GCF_000008865.2, GCF_000013425.1, GCF_000240185.1 were downloaded separately from NCBI RefSeq database [(Sayers et al. 2025)](https://paperpile.com/c/k3UvUm/BQtd). The high complexity regions were obtained from Pirogov et al. [(Pirogov et al. 2021)](https://paperpile.com/c/k3UvUm/RubGW). The VCF file of the germline mutations for the human pangenome was obtained from the Human Pangenome Reference Consortium (HPRC) (<https://s3-us-west-2.amazonaws.com/human-pangenomics/pangenomes/freeze/freeze1/minigraph-cactus/hprc-v1.1-mc-chm13/hprc-v1.1-mc-chm13.vcfbub.a100k.wave.vcf.gz>) [(Liao et al. 2023)](https://paperpile.com/c/k3UvUm/qm7p). Finally, the mutation rates in human genomes were downloaded from <http://genetics.bwh.harvard.edu/downloads/Vova/Roulette/> [(Seplyarskiy et al. 2023)](https://paperpile.com/c/k3UvUm/qvX3). All code is released under the MIT License.

“””
